# Predicting Stroke Risk Based on Health Behaviours: Development of the Stroke Population Risk Tool (SPoRT)

**DOI:** 10.1371/journal.pone.0143342

**Published:** 2015-12-04

**Authors:** Douglas G. Manuel, Meltem Tuna, Richard Perez, Peter Tanuseputro, Deirdre Hennessy, Carol Bennett, Laura Rosella, Claudia Sanmartin, Carl van Walraven, Jack V. Tu

**Affiliations:** 1 Ottawa Hospital Research Institute, Ottawa, Ontario, Canada; 2 Institute for Clinical Evaluative Sciences, Ottawa and Toronto, Ontario, Canada; 3 Statistics Canada, Ottawa, Ontario, Canada; 4 Department of Family Medicine, University of Ottawa, Ottawa, Ontario, Canada; 5 Epidemiology and Community Medicine, University of Ottawa, Ottawa, Ontario, Canada; 6 Bruyère Research Institute, Ottawa, Ontario, Canada; 7 Public Health Ontario, Toronto, Ontario, Canada; 8 Dalla Lana School of Public Health, University of Toronto, Toronto, Ontario, Canada; 9 Department of Medicine, University of Ottawa, Ottawa, Ontario, Canada; 10 Sunnybrook Schulich Heart Centre, University of Toronto, Toronto, Ontario, Canada; 11 Institute of Health Policy, Management, and Evaluation, University of Toronto, Toronto, Ontario, Canada; Indiana University School of Medicine, UNITED STATES

## Abstract

**Background:**

Health behaviours, important factors in cardiovascular disease, are increasingly a focus of prevention. We appraised whether stroke risk can be accurately assessed using self-reported information focused on health behaviours.

**Methods:**

Behavioural, sociodemographic and other risk factors were assessed in a population-based survey of 82 259 Ontarians who were followed for a median of 8.6 years (688 000 person-years follow-up) starting in 2001. Predictive algorithms for 5-year incident stroke resulting in hospitalization were created and then validated in a similar 2007 survey of 28 605 respondents (median 4.2 years follow-up).

**Results:**

We observed 3 236 incident stroke events (1 551 resulting in hospitalization; 1 685 in the community setting without hospital admission). The final algorithms were discriminating (C-stat: 0.85, men; 0.87, women) and well-calibrated (in 65 of 67 subgroups for men; 61 of 65 for women). An index was developed to summarize cumulative relative risk of incident stroke from health behaviours and stress. For men, each point on the index corresponded to a 12% relative risk increase (180% risk difference, lowest (0) to highest (9) scores). For women, each point corresponded to a 14% relative risk increase (340% difference, lowest (0) to highest (11) scores). Algorithms for secondary stroke outcomes (stroke resulting in death; classified as ischemic; excluding transient ischemic attack; and in the community setting) had similar health behaviour risk hazards.

**Conclusion:**

Incident stroke can be accurately predicted using self-reported information focused on health behaviours. Risk assessment can be performed with population health surveys to support population health planning or outside of clinical settings to support patient-focused prevention.

## Introduction

Stroke is the second leading cause of death worldwide.[1] The majority of people have multiple, largely preventable risks such as smoking, physical inactivity, poor diet, hypertension, obesity, and diabetes.[2] Discouragingly, risks such as physical inactivity and obesity are becoming more prevalent and other risks, such as poor diet, are not improving.[2]

All industrialized countries have clinical guidelines for targeted and evidence-based prevention of cardiovascular disease. These guidelines recommend assessment of cardiovascular risk using multivariable risk algorithms.[3, 4] For the most part, predictive stroke risk algorithms have focused on biophysical risks, such as hypertension, and disease risks, such as diabetes and atrial fibrillation.[3, 4]

Risk algorithms have also begun to be developed for population health purposes that typically do not include physical measures.[5] The main purpose of population risk algorithms, beyond describing the distribution of risk [6, 7], is to predict the number of people who will develop a disease or condition and to estimate the population burden of risks and the impact of health interventions. Population risk is calculated by applying the risk algorithm to current population health surveys. For many diseases, including diabetes and cardiovascular disease, the use of only self-reported risk exposures has been shown to have predictive accuracy that is comparable to risk algorithms that are created with risk exposures from physical measures.[8, 9]

It may be that algorithms based on self-reported risks can be developed for dual purposes of population and individual use. Increasingly, cardiovascular guidelines include recommendations for interventions that target unhealthy lifestyle and health behaviours, based on a patient’s risk of disease.[10, 11] As well, there is a move towards care that is community-based and patient centred. Patients are encouraged to participate in their own prevention, which may begin prior to, or in conjunction with, clinical care. A wide range of health behaviour interventions that effectively reduce the risk of stroke are available for both the pre-clinical and clinical settings but are underused.[12, 13]

Clinicians appear to favour health behaviour interventions over medications for low- and medium-risk patients [14], but existing cardiovascular risk algorithms seldom assess the role of health behaviours beyond smoking. This means that clinicians have difficulty communicating the degree to which health behaviours contribute to cardiovascular risk, as well as the potential benefit from lifestyle improvement. For example, two patients may have the same level of cardiovascular risk with considerably different behavioural risk factors. An older patient who is physically active, a non-smoker, and has a favourable diet may confer small or no benefit from further lifestyle modification. Conversely, a younger patient with the same cardiovascular risk who is physically inactive and has a poor diet may be motivated knowing the absolute and/or relative benefit of improving their lifestyle.[15]

We set out to examine whether stroke can be accurately predicted using self-reported information that focuses on health behaviours (smoking, physical activity, diet, alcohol consumption) and stress, independent of biophysical measurements (the **S**troke **Po**pulation **R**isk **T**ool [SPoRT]). We foresee three potential applications for developing such an algorithm: first, to facilitate decision-making for cardiovascular disease prevention through health behaviours; second, to estimate stroke risk in pre-clinical settings; and third, to allow estimation of stroke risk at the community level.

## Methods

This study was approved by the Ottawa Health Science Network Research Ethics Board (formerly the Ottawa Hospital Research Ethics Board).

### SPoRT derivation and validation cohorts

The derivation cohort consisted of 82 259 Ontario household respondents between the ages of 20 and 83 years from the combined 2001, 2003 and 2005 Canadian Community Health Surveys (CCHS [cycles 1.1, 2.1, and 3.1]), conducted by Statistics Canada.[16] The validation cohort consisted of respondents to the 2007/2008 CCHS survey (cycle 4.1).

These surveys, which used a multistage stratified cluster design that represented 98% of the Canadian population over the age of 12 years, attained an average response rate of 80.5%. The surveys were conducted through telephone and in-person interviews and all responses were self-reported. The details of the survey methods have been previously published.[16]

Consenting CCHS respondents who did not self-report a prior history of stroke were followed until incident stroke event, death, loss to follow-up (defined as loss of health care eligibility), or March 31, 2012. To ascertain stroke events, the CCHS respondents were individually linked to three population-based databases: 1) hospitalization records from the Canadian Institute for Health Information Discharge Abstract Database, 2) vital statistics (for cause of death—available only until Dec 31, 2009); and, 3) ambulatory physician records from the Ontario Health Insurance Program. Stroke events were ascertained using validated diagnostics codes and criteria. For hospitalized stroke, there was a 92% agreement between discharge diagnoses of stroke and chart reviews.[17] For stroke diagnosed in the hospital or community, the sensitivity was 68% and specificity 98.9%.[18] Diagnostic codes for stroke included TIA (unless otherwise specified) and followed the Canadian Stroke Network definition (ICD-9 codes: 362, 430, 431, 434, 435, 436; and ICD-10 codes: G45, H340, H34.1, I60, I61, I63, I64 excluding I608, I636, and G454 for most-responsible hospital diagnosis or underlying cause of death).[19] Stroke in the community setting were ascertained using similar ambulatory physician diagnoses (see Tu et al.[18] for details).

Across the three surveys, 99 929 Ontario CCHS respondents consented to health care follow-up. Respondents were excluded if they did not provide a valid universal health insurance program number (required for data linkage; n = 302), had suffered a stroke before the survey (n = 1 462), or were not aged between 20–83 years (n = 15 390). If a respondent was included in more than one CCHS cycle (n = 516), only their earliest survey response was included. The validation cohort consisted of 28 605 respondents after applying the same exclusion criteria (health insurance number n = 107; previous stroke n = 580; age n = 4 822; previous CCHS cycle n = 524).

### Risk factors for stroke

We selected and examined the association between incidence of stroke and each of the following risk factors: age, sex, four health behaviours (smoking, alcohol consumption, diet, and physical activity), stress, sociodemographic factors (ethnicity, immigration status, income [individual and family], education [individual and highest family education], neighbourhood deprivation), chronic conditions (self-report of physician-diagnosed diabetes, coronary heart disease, and hypertension), and body mass index (calculated from self-reported height and weight). See S1 Table for definitions of the risk factors considered.

### Model development

The primary outcome was incident stroke, resulting in hospitalization (study end-date March 31, 2012). There were five secondary outcomes: i) death from stroke; ii) death or hospitalization from stroke (study end-date for these two outcomes is December 31, 2009, reflecting the most recently available cause-specific mortality data); iii) hospitalized ischemic stroke only; iv) hospitalized stroke excluding TIA; and, v) stroke diagnosed in the community setting by a physician or resulting in a hospitalization. To increase statistical power, the secondary outcomes were assessed by combining the derivation and validation cohorts.

We used a Cox proportional hazards model to test the significance of each potential risk factor on the hazard of incident stroke. A competing risk approach was used for all analyses: all-cause death as a competing risk in the primary analyses and non-stroke death in the secondary analyses.[20–22] Time to stroke was calculated as the number of days from survey administration to admission date for incident stroke hospitalization or stroke death. Each exposure variable was centered on the cohort mean.

We created the models for males and females separately using a pre-specified stepwise approach that began with age, followed by health behaviours, sociodemographic indices, intermediate risk factors (such as body mass index) and proximal risks (such as self-reported diabetes, hypertension, and heart disease). Variables were added considering their ability to improve discrimination and calibration (as described below).

We included age with time interaction to address the proportional hazard assumption of traditional Cox models and to allow risk estimation for different follow-up times. We assessed age as a predictor using several different categorical and continuous forms, including spline functions.

We created an index that summarized behavioural risk factors to reflect the study’s focus on these factors. Typically, predictive risk indices are created after model development to facilitate interpretation by the general user. We generated the index of behavioural risk factors—called the SPoRT Behaviour Score—during model development to increase statistical and discriminating power when examining multiple behavioural risk factors and categories.[23] This process also supported the creation of a model structure that lessened the potential for intermediate and proximal risk factors to reduce the association between behavioural risk factors and stroke.[24–27] For example, we would expect a reduced effect size of diet and physical activity if BMI and diabetes were simultaneously included in the model without considering that BMI and diabetes are risk factors on the causal pathway between health behaviours and stroke.

The SPoRT Behaviour Score was created through the following steps. First, the hazards for individual risk factors were examined using a reference group of respondents with the most favourable behaviour for all risk factors. Age-adjusted hazards for each risk factor and exposure category were rank-ordered and scores assigned based on the estimated hazard ratios. The scores were then rounded to integer values while maintaining the initial rank order of hazards to minimize the difference in observed versus predicted number of events—overall and in predefined subgroups (see Assessment of predictive accuracy)—while preserving the initial rank-order of the respective risk factor scores.[23]

Next, we added intermediate and proximal risk factors to the model, assessing hazards and improvement in predictive accuracy. We assessed interaction terms, focusing on age and behavioural risks as well as interaction between behavioural risk factors. (see S2 Table for details).

The prevalence of missing values was less than 5% for any variable. In order to estimate a SPoRT Behaviour Score for each subject, missing values for behavioural risk factors were imputed based on mean values for the respondent’s age, sex and local health region.[28] Missing values for other risk factors were maintained as separate categories to allow future application of the algorithm for other similar population health surveys.

### Assessment of predictive accuracy

We sought to develop a predictive algorithm that was both well calibrated and discriminating, with an emphasis on calibration for behavioural risks and use in the community setting.[29]

Calibration is the ability of an algorithm’s predictive estimates to closely approximate observed risk or to correctly rank subjects' risk.[30] We compared predicted to observed risk for the overall population, as well as across predefined subgroups (67 subgroups for males and 65 for females) identified as being important to clinicians and policy actors through a structured consultation process.[31] Calibration subcategories included: all behavioural risk categories, deciles of risk, age groups, health planning regions, sociodemographic groups, body mass index, hypertension status, and diabetes status. We predefined an important difference in calibration as a relative difference of greater than 20% between observed and predicted estimates for those categories with more than 5% of total stoke cases.[31]

Discrimination is the ability to differentiate individuals at high risk from those at low risk.[30] We assessed the C-statistic and 75:25 and 95:5 risk percentile ratios for survival data with time-dependent covariates.[32] Further details of the methods are provided in S2 Table.[33]

## Results

Baseline characteristics of the study cohorts are presented in S3 Table. The derivation cohort had a median age of 48.2 for males and 49.4 for females and a median follow-up time of 8.6 years, representing 688 000 person years. Overall, 1 551 incident stroke hospitalizations were observed (1.09% 5-year risk), of which 709 occurred in males (1.15% 5-year risk) and 842 in females (1.04% 5-year risk). There were an additional 50 out-of-hospital deaths due to incident stroke and an additional 1 685 strokes that occurred in the community setting (2.4% 5-year risk 2.5% for males and 2.4% for females).

**Fig 1:**
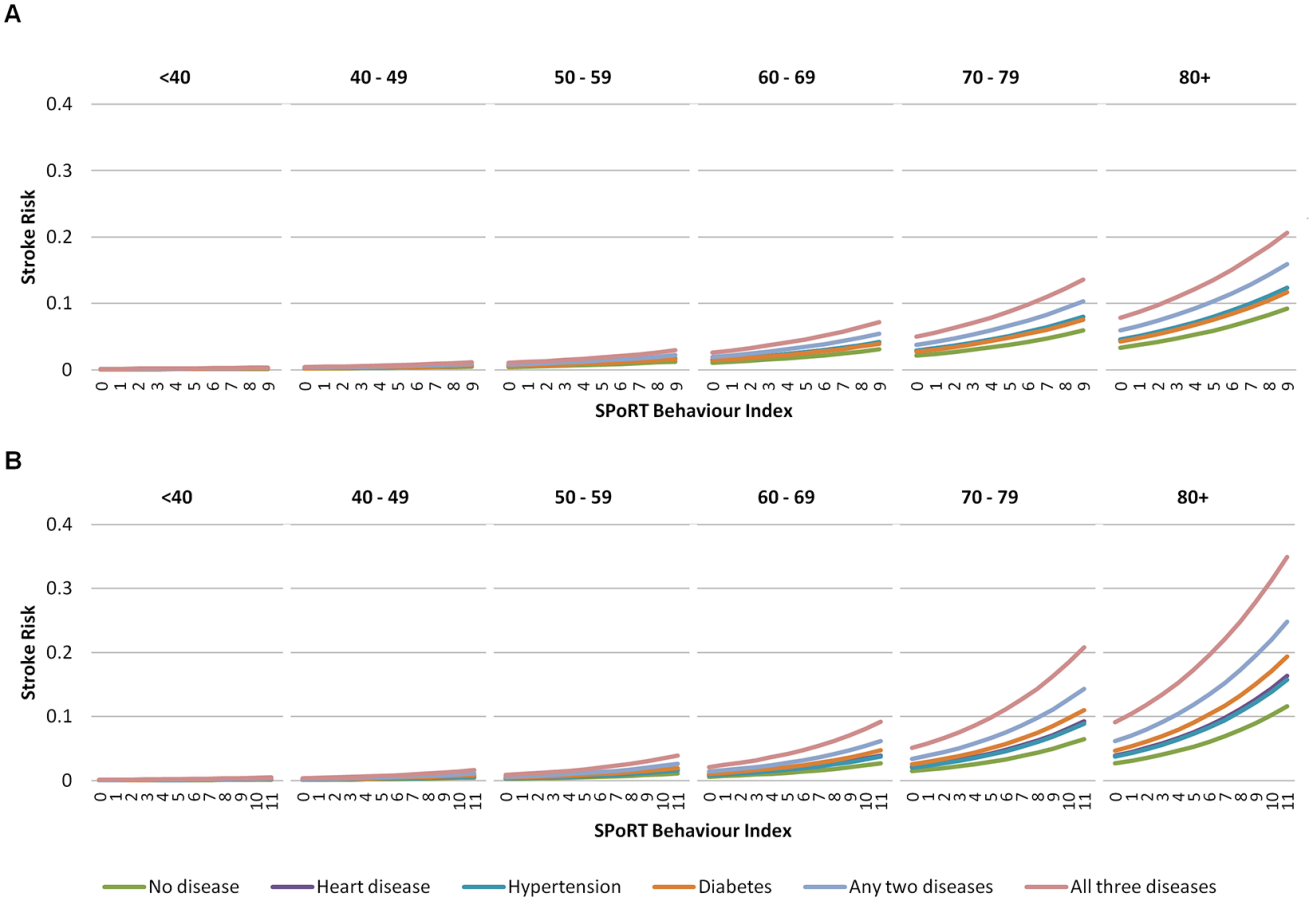
Predicted 5-year risk of stroke by age group and SPoRT behavioural index value.

The sex-specific index of behavioural risk is shown in Table 1 (see S4 Table for the hazards of individual risks). In the final model, each point on the SPoRT Behaviour Score corresponded to a 12% increase in stroke for men (180% risk difference from lowest (0) to highest (9) scores) and a 14% increase in stroke for women (340% difference from lowest (0) to highest (11) scores) (Fig 1). Men had increases in stroke risk of 37% for previously diagnosed hypertension (women, 39%), 36% for heart disease (women, 44%) and 29% for diabetes (women, 74%). Men with all three chronic conditions and maximum scores for all behavioural risks had a 560% increased risk of stroke compared to men with no risk factors present (no poor health behaviours and no chronic conditions) (1400% for women).

**Table 1:** SPoRT index of health behaviour and stress.

Risk Factor*	Description	Male index	Female index
Smoking			
Heavy smoker	Daily current smoker (≥1 pack/day)	3	4
Light smoker	Daily current smoker (<1 pack/day)	2	3
Former smoker	Former daily smoker	1	1
*Non-smoker*	*Former occasional smoker or never smoker*	*0*	*0*
Alcohol			
Heavy drinker	>21 (men) or >14 (women) drinks/week in previous month or weekly bingeing behaviour^†^	1	2
*Moderate drinker*	*5 to 21 (men) or 3 to 14 (women) drinks/week*	*0*	*0*
Light drinker	0 to 4 (men) or 0 to 2 (women) drinks/week	0	1
Occasional drinker	<1 drink/month	0	1
Current non-drinker	No alcohol consumption in the last 12 months	1	2
Physical activity			
Inactive	0 to <1.5 METs/day^‡^	2	1
Moderately active	1.5 to <3 METs/day	1	0
*Active*	≥*3 METs/day*	*0*	*0*
Diet			
Poor diet	<7 weekly fruit and vegetable serving	2	2
Fair diet	7 to <14 weekly fruit and vegetable serving	1	1
*Adequate diet*	≥ 14 weekly fruit and vegetable serving	*0*	*0*
Stress			
High stress	Self-perceived stress: ‘quite a bit’ or ‘extremely’	1	2
*Low stress*	*Self-perceived stress*: *‘not at all’*, *‘not very’*, *or ‘a bit’*	*0*	*0*
**Maximum score**		9	11

Each point on the SPoRT Behaviour Score increases stroke risk by 12% for males or 14% for females (see full model on Table 2). The maximum score for males equals a 180% (9 x 12%) risk difference compared to the lowest score; 340% (11 x 14%) for females.

*Reference group is in italics.

^†^ Bingeing was defined as ≥5 drinks/day on any occasion.

^‡^METs are Metabolic Equivalent of Task (kcal/kg/day). For example, the “inactive” physical activity is equal to walking for exercise less than 30 min per day (3 METS/hr).

**Fig 2:**
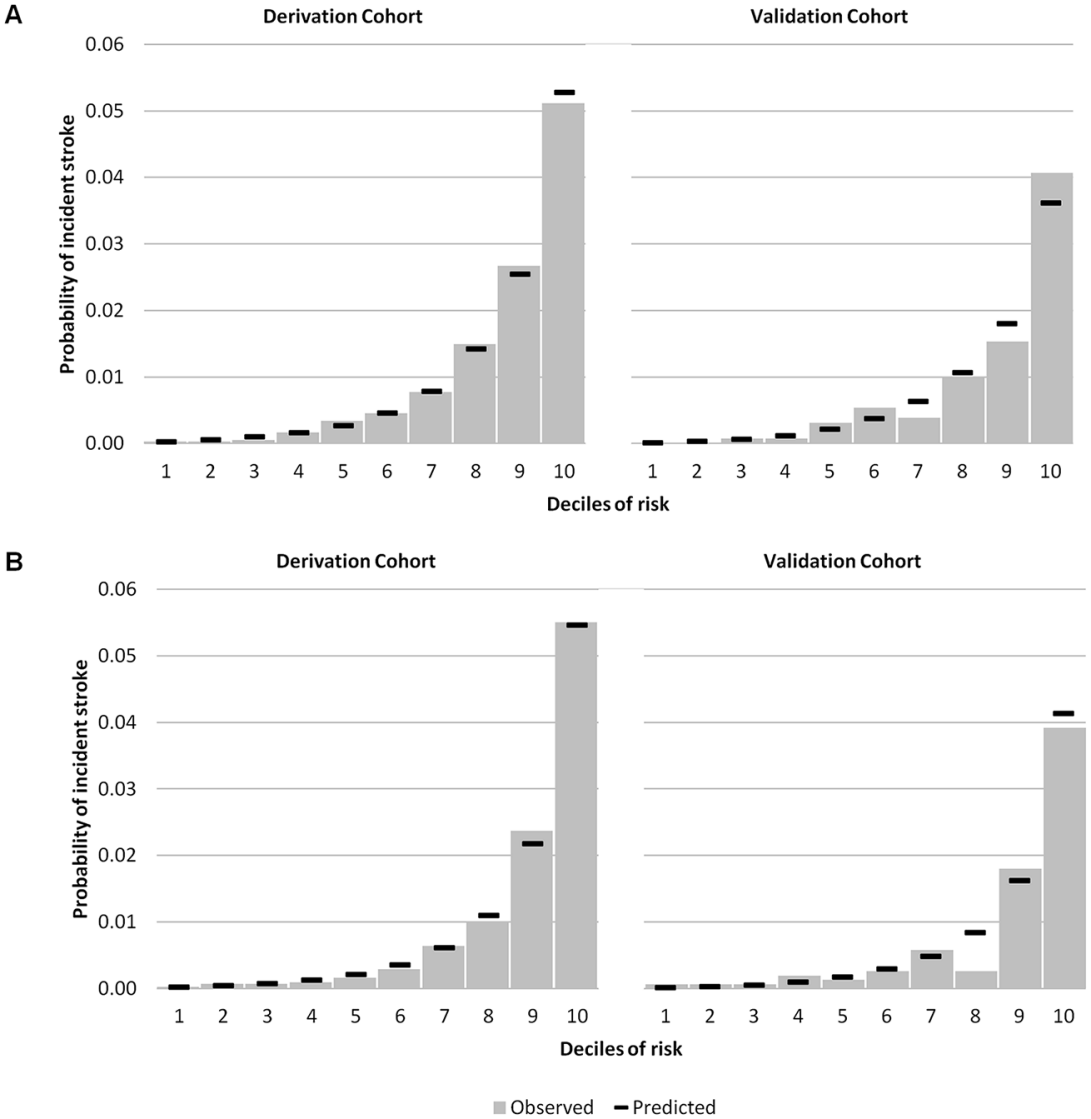
Observed versus predicted risk of 5-year incident stoke by risk decile—derivation and validation cohorts. Panel A = males; Panel B = females. *Statistically significant difference between observed and predicted risk.

**Fig 3:**
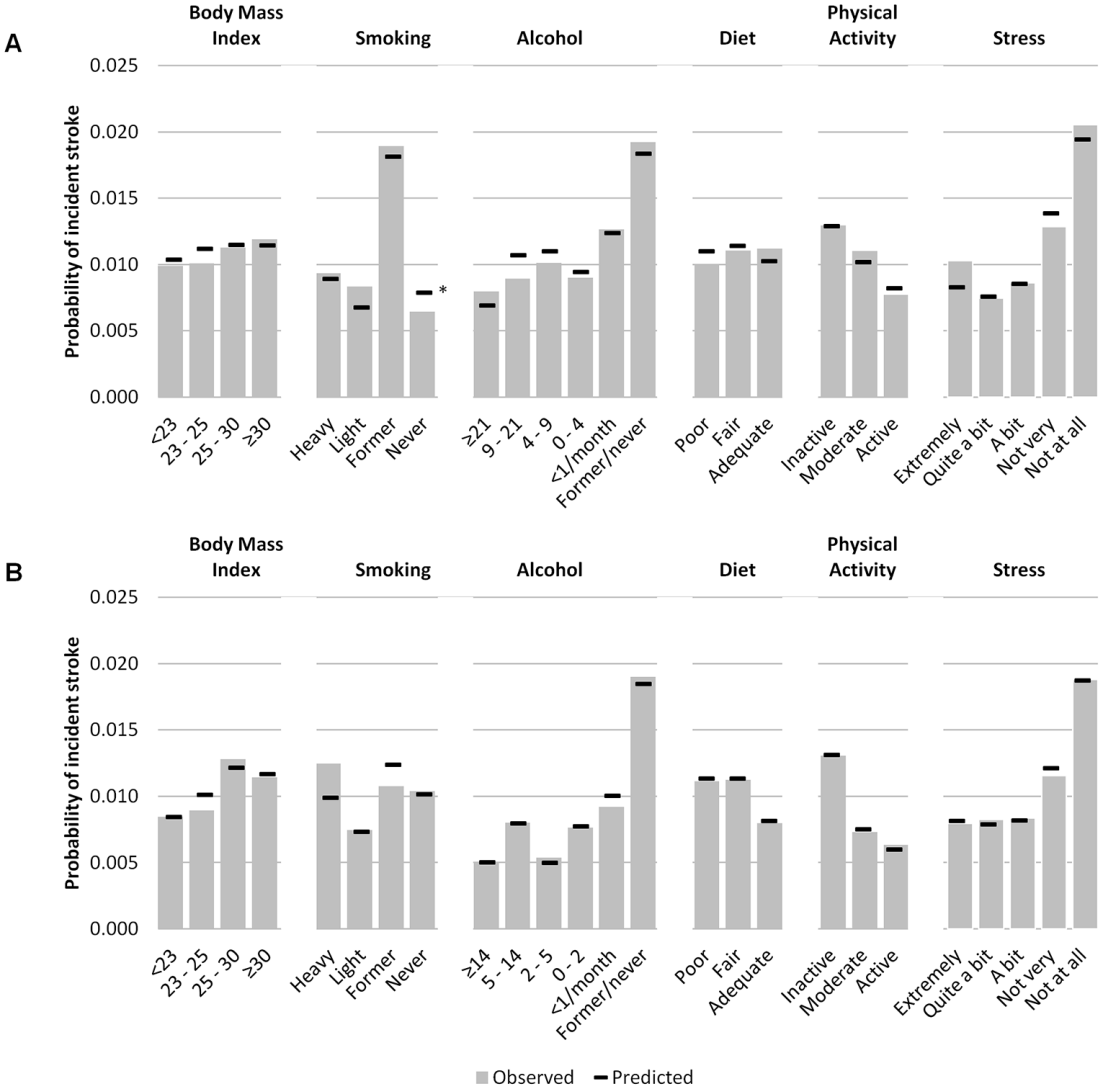
Observed versus predicted risk of 5-year incident stoke by health behaviour, BMI, and stress. Panel A = males; Panel B = females.

Table 2 presents the hazards and performance for SPoRT. The final model C-statistic, assessing discrimination, was 0.85 (95% CI 0.83–0.86) for males and 0.87 (95% CI 0.85–0.88) for females. Calibration/accuracy improved between the age-only and final models with a less evident change in discrimination. Using age as the only predictor, 22 of 67 predefined subgroups for males showed greater than 20% difference between the predicted and observed stroke events, which reduced to 2 subgroups in the final model (4 of 65 groups for the female model). Figs 2 and 3 show predicted and observed risk by deciles and details of calibration for the behavioural risk factors. Table 2 shows the overall observed and predicted risk, including a summary for calibration by subgroup. S1 and S2 Figs summarize risk as nomograms. We have also created an individual stroke risk calculator which is available online at www.projectbiglife.ca.

**Table 2:** Stroke Population Risk Tool (SPoRT)–Model.

	Hazard Ratio (95% CI)	
	Male Model*	Female Model*
Age	1.11 (1.09–1.13)	1.11 (1.09–1.12)
Age spline (65 years)	0.97 (0.95–0.99)	
Age time (per year)	0.997 (0.995–0.9999)	0.996(0.993–0.997)
SPoRT Behaviour Score** (per unit)	1.12 (1.07–1.17)	1.15 (1.11–1.19)
Hypertension		
No	1.0 [Reference]	1.0 [Reference]
Yes	1.37(1.16–1.60)	1.39 (1.20–1.61)
Missing	0.80 (0.11–5.88)	1.53 (0.24–9.84)
Heart Disease		
No	1.0 [Reference]	1.0 [Reference]
Yes	1.36 (1.14–1.63)	1.44 (1.22–1.71)
Diabetes		
No	1.0 [Reference]	1.0 [Reference]
Yes	1.29 (1.06–1.57)	1.74 (1.45–2.09)
Missing	–-	
Survey cycle		
3.1 (2005)	1.0 [Reference]	1.0 [Reference]
2.1 (2003)	1.03 (0.85–1.26)	1.05 (0.87–1.26)
1.1 (2001)	1.26 (1.04–1.53)	1.18 (0.99–1.42)
**Model Assessment**		
Discrimination		
C-stat (95% CI)	0.85 (0.83–0.86)	0.87 (0.85–0.88)
Ratio of 75 to 25 risk percentile (5-year risk range)	13.3 (0.11 to 1.40)	14.0 (0.08 to 1.07)
Ratio of 95 to 5 risk percentile	149.7 (0.03 to 4.79)	179.2 (0.026 to 4.70)
Calibration		
Subgroup differences No. (%)	2 (3.0)^†^	4 (5.7) ^‡^

*The full model was calibrated to survey cycle year

**0–9 for males, 0–11 for females

^†^ Observed versus predicted estimates were compared for 67 subgroups—selected based on meeting the criteria of having more than 5% of total observed stroke events (i.e., more than 22 events). We report the number of subgroups where there was a clinically important difference (predefined as ≥ 20% difference) in observed versus predicted number of events. The 67 subgroups were: deciles of predicted risk (4), local health networks (9), age (7), body mass index (4), physical activity (3), alcohol consumption (6), smoking (4), diet (3), self perceived stress (4), ethnicity (1), family income (7), family education (4), high blood pressure (2), diabetes (2), heart disease (2) SPoRT Behaviour Score (5)

^‡^ Observed versus predicted estimates were compared for 65 subgroups—selected based on meeting the criteria of having more than 5% of total observed stroke events (i.e., more than 23 events). We report the number of subgroups where there was a clinically important difference (predefined as ≥ 20% difference) in observed versus predicted number of events. The 65 subgroups examined were: deciles (4), local health networks (8), age (6), body mass index (5), physical activity (3), alcohol consumption (5), smoking (4), diet (3), self perceived stress (4), ethnicity (1), family income (7), family education (3), high blood pressure (2), diabetes (2), heart disease (2), SPoRT Behaviour Score (6).

The validation cohort showed similar discrimination and calibration compared to the development data (see Fig 2 for predictive and observed risk for the validation cohort by decile). The C-statistic in the validation cohort was 0.85 (95% CI 0.81–0.88) for males and 0.85 (95% CI 0.81–0.89) for females. The overall predicted risk for the follow up period in the validation cohort was 0.799% for males compared to 0.798% observed risk (relative difference is almost null). For females, the relative difference was 6.8% (0.78% versus 0.73%).

**Fig 4:**
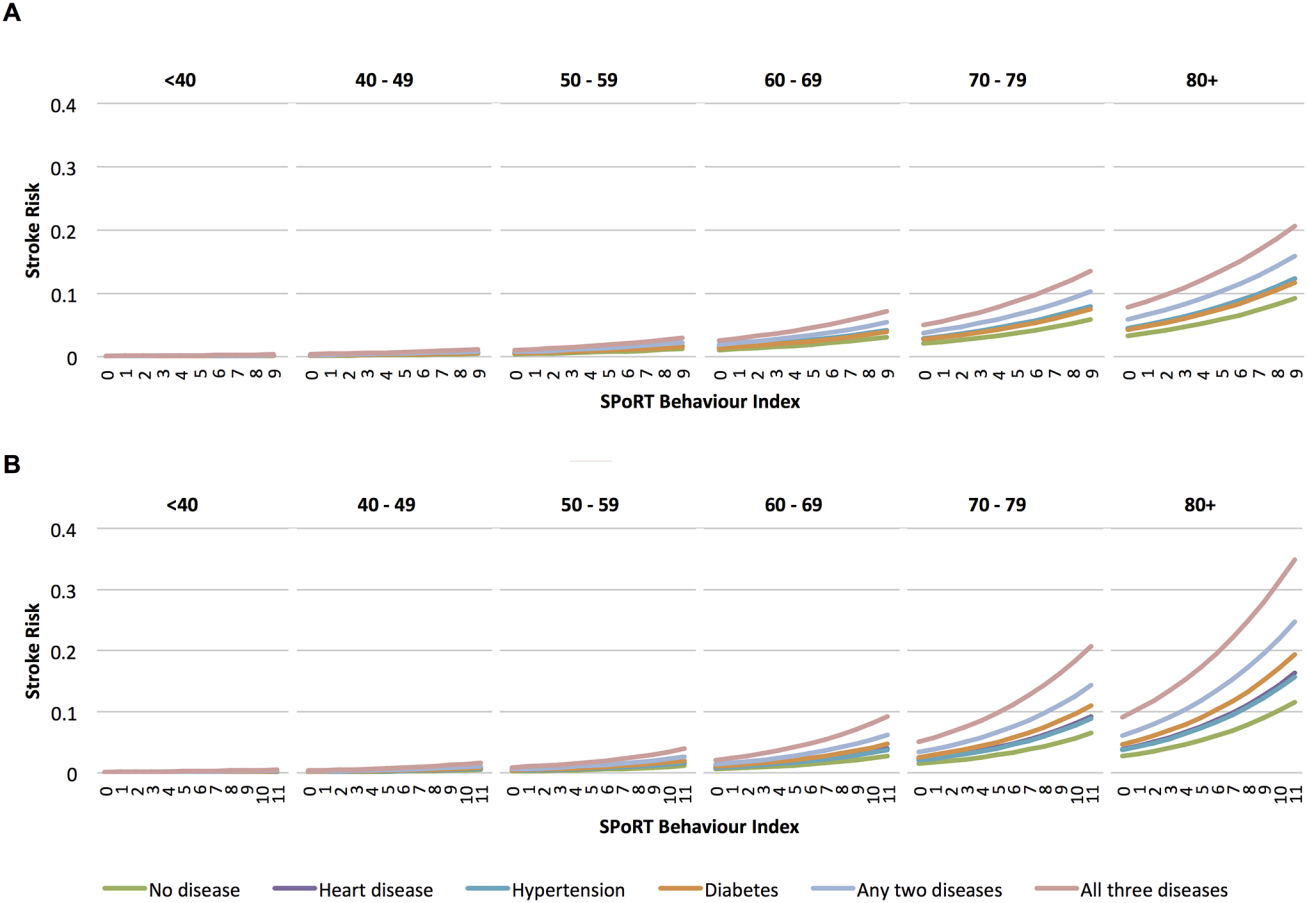
Secondary analysis—observed (O) versus predicted (P) risk of 5-year incident stoke by risk decile, combined development and validation cohorts. Panel A = males; Panel B = females. Abbreviations: O = observed; P = predicted. *Primary outcome.

SPoRT for secondary stroke outcomes had similar risk hazards with a trend toward higher risk hazards for health behaviours in more severe (hospitalized stroke without TIA) or discrete (ischemic stroke only) outcomes (Fig 4 and S5 Table).

## Discussion

This study demonstrated that stroke risk can be accurately predicted solely using self-responses from population health surveys that focus on health behaviours. A study strength was development and validation of the algorithms using a large population-based cohort. We were able to include a large number of predictive risks and subgroups while minimizing the risk of over-fitting, thereby maintaining generalizability.

SPoRT accurately predicted risk for over 130 risk groups: including people exposed and not exposed to unhealthy behaviours, other more proximal risks, and risks that were not included in the final model (e.g., BMI). SPoRT had equally high predictive accuracy for risk deciles in an external validation cohort and similar performance for a range of outcomes, including stroke diagnosed in the hospital or community. The relative importance of behavioural risks and their level of effect, as described in the SPoRT Behaviour Score, were similar to epidemiology studies.[25]

### Implications for public health, community and clinical prevention

The SPoRT algorithm complements other approaches to stroke risk assessment by informing public health planners, patients and clinicians about the contribution of health behaviours. Clinical guidelines from the World Health Organization and most countries recommend a graded approach to cardiovascular disease prevention that includes interventions with low individual cost targeting the entire population combined with individual therapy tailored to risk levels.[34, 35] A graded prevention approach is best accompanied by a graded assessment of cardiovascular risk, which starts with simple and accessible assessment of as wide a target population as possible, followed by progressively more intensive risk assessment to discriminate among individuals with progressively less prevalent (but clinically important) risk factors. Ideally, each stage of risk assessment supports corresponding interventions for that setting.

In the public health setting, where risk assessment involves use of population health surveys to ascertain risk exposure and population diffusion of risk, multivariable risk algorithms have been shown to be the most discriminating approach.[5] Our study suggests that risk of stroke can be discriminately assessed using population health surveys and multivariable risk algorithm; and, that stroke risk is concentrated in the elderly and in groups with multiple risk factors.

In the general population, risk assessment is performed by individuals in the community and focuses on health behaviours and other risk factors that are common, contribute to a large burden of disease, and are modifiable in the community setting. That is not to say that SPoRT should replace other clinical algorithms that include measurement of blood pressure and lipids. Rather, we suggest a graded approach to risk assessment that begins in the community setting and focuses on health behaviours. In the primary care setting, risk stratification includes blood pressure, lipids and other more detailed risk information, which potentially improves risk stratification and supports decision-making about medication. In the speciality setting, progressively more intensive risk assessment corresponds to more intensive treatment options.

### Opportunities in public health and international settings

Assessing population risk is useful for planning purposes, including predicting future disease incidence and assessing the effectiveness of community-wide prevention strategies.[5] Few jurisdictions have population data that contains the clinical and biophysical measures required for application of clinic CVD risk algorithms. However, many jurisdictions have self-reported health surveys that could be used to estimate risk using SPoRT or similar risk algorithms. Furthermore, SPoRT’s population-based focus enables several approaches for validation, recalibration and application that are not typically available to clinical risk algorithms.[5] For example, population health surveys from other countries can be used to recalibrate SPoRT based on the population-specific prevalence and distribution of risk factors. SPoRT risk estimates can be further calibrated by adjusting predicted population estimates against observed population stroke incidence.[36]

### Current study in perspective

To our knowledge, SPoRT is the only cardiovascular risk algorithm that can be applied to population health surveys. As well, we are aware of only one other cardiovascular algorithm that includes all major behavioural risks.[9] History of hypertension, diabetes, and heart disease are included in SPoRT but only as self-reported measures rather than clinical measures or confirmed diagnosis. Despite this constraint, SPoRT has predictive accuracy as high, if not higher, than risk algorithms that rely on clinical measures.[4]

We purposefully emphasized the role of health behaviours over proximal risks, such as hypertension and diabetes, to facilitate prevention. By first including behavioural risks and summarizing these risks as the SPoRT Behaviour Score we created a simple hierarchical structure that preserves the contribution of behaviours to stroke risk. If our sole purpose was predicting stroke, rather than predicting stroke based on health behaviours, we would likely have found that behavioural risks have little additional prognostic ability over a smaller selection of traditionally included proximal risks (e.g., measured blood pressure). Furthermore, our emphasis on calibration informs how well SPoRT performs in assessing stroke risk based on behaviours.

There are other cardiovascular indices that summarize preventable risks, such as the index of *Ideal Cardiovascular Health* (ICH) developed by the American Health Association.[35] Uniquely, SPoRT can express individual health behaviours as either a relative or absolute stroke risk versus a categorical scale (see S6 Table for a comparison of SPoRT and ICH). Despite stroke being a leading cause of morbidity, many people in community setting will likely interpret large relative differences in stroke risk (over 500% relative difference in stroke risk across people of the same age) differently knowing that the baseline risk of stroke is low (1.09% 5-year risk in our Ontario population, 5 to 95% range 0.03 to 4.62%).

### Limitations

The chief limitation of this study is potential misclassification error resulting from the exclusive use of self-reported risks and routinely-collected stroke data. While more accurate risk factor ascertainment could improve discrimination and calibration, SPoRT already has a high discrimination and favourable calibration. Other studies have also found that chronic diseases can accurately be assessed using self-reports. Gaziano et al. and Qiao et al. showed there are only modest classification differences when CVD risk assessment is performed with and without clinical and laboratory measures.[37, 38] As well, there are many diabetes risk algorithms developed to ascertain risk outside the clinic setting using only self-reported measures.[39] Furthermore, the most influential risk factors in SPoRT are extensively used and validated world-wide: there have been favourable studies for self-reported smoking status validated against urine cotinine levels, and heart disease, hypertension and diabetes validated against physician diagnoses.[40, 41] Thus, using physician-diagnosed disease or urine test for smoking would not improve stroke risk discrimination or accuracy. Similarly, self-reported height and weight were used to estimate BMI. Validation studies for the CCHS have confirmed a modest misclassification of self-reported BMI compare to measured BMI and correction factors are available.[42] We did not use those correction factors for two reasons: first, modest reclassification of BMI will have a small influence on predictive risk, given the BMI risk occurred only at high BMI levels (BMI 35+). Second, the main indented use of SPoRT is for population health surveys without measured BMI and self-reported use in the community setting. This means that our use of self-reported BMI is consistent in both development, validation and application, thus ensuring appropriate calibration. Regardless, it will be important to assess SPoRT in other external populations—particularly since our external validation population was similar to the derivation population.

There is a greater degree of misclassification error for alcohol consumption, physical activity and diet; however, it is reassuring that we found the measures used in our study are discriminating and have a similar association with stroke as seen in other studies. For alcohol, there are concerns that self-reports considerably underreport consumption. That stated, there is consistent evidence of a “J” shape relationship of hazards that was replicated in our study.[43] Physical activity has modest self-reported ascertainment accuracy compared to accelerometer measures, with about half of the respondent of self-reported surveys accurately reporting their activity and others equally over- and under-reporting activity up to 30 minutes per day.[44]

Diet, likely the most challenging CVD risk to ascertain using brief self-reports, is important to consider in risk assessment for at least three reasons: there is a clear and important relationship between diet and CVD; a high proportion of people in many countries have poor diet quality; and, diet is potentially modifiable with corresponding improvement in CVD risk.[45, 46] Increasingly, there is emphasis to ascertain overall diet quality rather than specific food types or nutrients. General population health surveys, such as the one used for our study cohort, use fruit and vegetable consumption as a proxy for overall diet quality. While there is modest over-report of fruit and vegetable consumption compared to repeated 24-hour food recall, there is good rank-order correlation between those that have high or low consumption of fruit and vegetables and overall diet quality.[46] Similar to previous studies examining all-cause mortality and all-cause hospitalization, we found that high potato and fruit juice consumption were hazardous for stroke risk and accordingly modified a brief dietary quality index.[47, 48] There is the potential for brief diet quality indices to have poor generalizability across jurisdictions. However, given the favourable predictive accuracy and a hazard that corresponds with the diet/CVD relationship seen in other studies, we believe that our study demonstrates the utility of brief self-reported diet questions for CVD risk assessment. Future studies should validate our (and other) brief diet quality measure for risk prediction.

The large study cohort and use of routinely-collected data meant that it was not feasible to individually verify the stroke events. However, we used identification approaches that have been shown to accurately ascertain stroke events.[17, 18] Moreover, we examined five different stroke outcomes using three different databases, with SPoRT showing equal predictive accuracy regardless of stroke endpoint. As expected, there was a small trend toward a lower hazard for the SPoRT Behaviour Score with a stroke definition that was broader and more heterogeneous.

Finally, our approach to create a hierarchical structure for the predictive algorithm, through the creation of the behavioural index, has limitations due to the early examination of the hazards of each behavioural risk. Current recommendations for prognostic algorithms recommend a more rigorous pre-specified approach that minimizes examination of outcome relationships when making decisions about predictor selection and form. In general, we had a high adherence to recommended algorithm development (see S2 Table) but allowed ourselves to deviate from recommendations, recognizing that development of algorithms for the population setting differs from the more common development in the clinical setting.[5, 49] For example, the large sample size and power of our study should reduce the risk of type 1 error compared to most clinical algorithms that use a much smaller sample of respondents. That said, in the future we plan to disaggregate the task of prognosis from etiognosis by developing a purely prognostic algorithm (ignoring causal pathways) and then separately perform analyses to estimate a hazard of risks from a causal perspective.[50]

### Conclusion

Stroke risk can be accurately predicted solely using information on health behaviours and other self-reported risks. SPoRT does not require clinical or laboratory data, making it well-suited for application using population health surveys as well as easy to implement for general population use in the community setting. The focus on health behaviours further facilitates patient-centred and population approaches for stroke and cardiovascular disease prevention.

## Supporting Information

S1 Fig Males: 5-year risk of hospitalized stroke based on behavioral and other risk factors. (DOCX)

S2 Fig Females: 5-year risk of hospitalized stroke based on behavioral and other risk factors. (DOCX)

S1 Table Definitions for exposure variables. (DOCX)

S2 Table Checklist for reporting clinical prediction research. (DOCX)

S3 Table Baseline characteristics of the derivation (CCHS 1.1 –CCHS 3.1) and validation (CCHS 4.1) cohorts. (DOCX)

S4 Table Hazard ratios for individual risk factors. (DOCX)

S5 Table Sensitivity Analysis. (DOCX)

S6 Table Comparison of SPoRT to Ideal Cardiovascular Health, developed by the American Heart Association. (DOCX)

S7 Table SPoRT formula. (DOCX)

S8 Table Crude and age standardized stroke incidence rate per 10000 person-years. (DOCX)

## References

[1] Lozano R, Naghavi M, Foreman K, Lim S, Shibuya K, Aboyans V et al: Global and regional mortality from 235 causes of death for 20 age groups in 1990 and 2010: a systematic analysis for the Global Burden of Disease Study 2010. The Lancet 2012, 380(9859):2095–2128. 10.1016/S0140-6736(12)61728-0 PMC10790329 23245604

[2] Lim SS, Vos T, Flaxman AD, Danaei G, Shibuya K, Adair-Rohani H et al: A comparative risk assessment of burden of disease and injury attributable to 67 risk factors and risk factor clusters in 21 regions, 1990–2010: a systematic analysis for the Global Burden of Disease Study 2010. The Lancet 2012, 380(9859):2224–2260. 10.1016/S0140-6736(12)61766-8 PMC4156511 23245609

[3] Manuel DG. The effectiveness of national guidelines for preventing cardiovascular disease: integrating effectiveness concepts and evaluating guidelines' use in the real world. Curr Opin Lipidol 2010, 21(4):359–365. 20581675 10.1097/MOL.0b013e32833c1f2b

[4] Ferket BS, Colkesen EB, Visser JJ, Spronk S, Kraaijenhagen RA, Steyerberg EW et al: Systematic review of guidelines on cardiovascular risk assessment: Which recommendations should clinicians follow for a cardiovascular health check? Arch Intern Med 2010, 170(1):27–40. 10.1001/archinternmed.2009.434 20065196

[5] Manuel DG, Rosella LC, Hennessy D, Sanmartin C, Wilson K. Predictive risk algorithms in a population setting: an overview. J Epidemiol Community Health 2012, 66:859–865. 10.1136/jech-2012-200971 22859516

[6] Rose G. Rose's Strategy of Preventive Medicine. Oxford: Oxford University Press; 2008.

[7] Manuel DG, Lim J, Tanuseputro P, Anderson GM, Alter DA, Laupacis A et al: Revisiting Rose: strategies for reducing coronary heart disease. British Medical Journal 2006, 332(7542):659–662. 16543339 10.1136/bmj.332.7542.659 PMC1403258

[8] Rosella LC, Manuel DG, Burchill C, Stukel TA, PHIAT-DM team: A population-based risk algorithm for the development of diabetes: development and validation of the Diabetes Population Risk Tool (DPoRT). J Epidemiol Community Health 2011, 65(7):613–620. 10.1136/jech.2009.102244 20515896 PMC3112365

[9] Chiuve SE, Cook NR, Shay CM, Rexrode KM, Albert CM, Manson JE et al Lifestyle‐Based Prediction Model for the Prevention of CVD: The Healthy Heart Score. Journal of the American Heart Association 2014, 3(6). 10.1161/JAHA.114.000954 PMC4338684 25398889

[10] Rabar S, Harker M, O'Flynn N, Wierzbicki AS, Guideline Development Group: Lipid modification and cardiovascular risk assessment for the primary and secondary prevention of cardiovascular disease: summary of updated NICE guidance. BMJ 2014, 349:g4356 10.1136/bmj.g4356 25035388

[11] Stone NJ, Robinson JG, Lichtenstein AH, Bairey Merz CN, Blum CB et al: 2013 ACC/AHA guideline on the treatment of blood cholesterol to reduce atherosclerotic cardiovascular risk in adults: a report of the American College of Cardiology/American Heart Association Task Force on Practice Guidelines. Circulation 2014, 129(25 Suppl 2):S1–45. 10.1161/01.cir.0000437738.63853.7a 24222016

[12] Lim SS, Gaziano TA, Gakidou E, Reddy KS, Farzadfar F, Lozano R et al: Prevention of cardiovascular disease in high-risk individuals in low-income and middle-income countries: health effects and costs. Lancet 2007, 370(9604):2054–2062. 18063025 10.1016/S0140-6736(07)61699-7

[13] Vos T, Carter R, Barendregt J, Mihalopoulos C, Veerman L, Magnus A et al: Assessing Cost-Effectiveness in Prevention. The University of Queensland, Brisbane, and Deakin University, Melbourne 2010.

[14] Schulte JM, Rothaus CS, Adler JN. Starting Statins—Polling Results. *New England Journal of Medicine* 2014, 371(4):e6 10.1056/NEJMclde1407177 25054734

[15] Sheridan SL, Viera AJ, Krantz MJ, Ice CL, Steinman LE, Peters KE et al: The effect of giving global coronary risk information to adults: a systematic review. Archives of internal medicine 2010, 170(3):230 10.1001/archinternmed.2009.516 20142567

[16] Beland Y. Canadian Community Health Survey—Methodological Overview. Health Reports 2002, 13(2):9–14. 12743956

[17] Kokotailo RA, Hill MD. Coding of stroke and stroke risk factors using international classification of diseases, revisions 9 and 10. Stroke 2005, 36(8):1776–1781. 10.1161/01.STR.0000174293.17959.a1 16020772

[18] Tu K, Wang M, Young J, Green D, Ivers NM, Butt D et al: Validity of administrative data for identifying patients who have had a stroke or transient ischemic attack using EMRALD as a reference standard. Can J Cardiol 2013, 29(11):1388–1394. 10.1016/j.cjca.2013.07.676 24075778

[19] CSS Information & Evaluation Working Group: Canadian Stroke Strategy Core Preformance Indicator Update 2010. In: Candain Stroke Network 2010: 1–7.

[20] Pintilie M. Dealing with competing risks: testing covariates and calculating sample size. Stat Med 2002, 21(22):3317–3324. 12407674 10.1002/sim.1271

[21] Gooley TA, Leisenring W, Crowley J, Storer BE. Estimation of failure probabilities in the presence of competing risks: new representations of old estimators. Stat Med 1999, 18(6):695–706. 10204198 10.1002/(sici)1097-0258(19990330)18:6<695::aid-sim60>3.0.co;2-o

[22] Pencina MJ, D'Agostino RB Sr., Larson MG, Massaro JM, Vasan RS. Predicting the 30-year risk of cardiovascular disease: the framingham heart study. Circulation 2009, 119(24):3078–3084. 10.1161/CIRCULATIONAHA.108.816694 19506114 PMC2748236

[23] Nardo M, Saisana M, Saltelli A, Tarantola S, Hoffman A, Giovannini E. Handbook on constructing composite indicators: methodology and user guide. In.: OECD publishing; 2005.

[24] Cecchini M, Sassi F, Lauer JA, Lee YY, Guajardo-Barron V, Chisholm D. Tackling of unhealthy diets, physical inactivity, and obesity: health effects and cost-effectiveness. Lancet 2010, 376(9754):1775–1784. 10.1016/S0140-6736(10)61514-0 21074255

[25] O'Donnell MJ, Xavier D, Liu L, Zhang H, Chin SL, Rao P et al: Risk factors for ischaemic and intracerebral haemorrhagic stroke in 22 countries (the INTERSTROKE study): a case-control study. The Lancet 2010, 376(9735):112–123. 10.1016/S0140-6736(10)60834-3 20561675

[26] Schisterman EF, Cole SR, Platt RW: Overadjustment bias and unnecessary adjustment in epidemiologic studies. Epidemiology 2009, 20(4):488–495. 19525685 10.1097/EDE.0b013e3181a819a1 PMC2744485

[27] Bansal A, Pepe MS. When does combining markers improve classification performance and what are implications for practice? Stat Med 2013, 32(11):1877–1892. 10.1002/sim.5736 23348801 PMC3893148

[28] Dalton AR, Bottle A, Soljak M, Okoro C, Majeed A, Millett C. The comparison of cardiovascular risk scores using two methods of substituting missing risk factor data in patient medical records. Informatics in primary care 2011, 19(4):225–232. 22828577 10.14236/jhi.v19i4.817

[29] Diamond GA. Future imperfect: the limitations of clinical prediction models and the limits of clinical prediction. Journal of the American College of Cardiology 1989, 14(3 Suppl A):12A–22A. 2768728 10.1016/0735-1097(89)90157-5

[30] Tripepi G, Jager KJ, Dekker FW, Zoccali C. Statistical methods for the assessment of prognostic biomarkers(part II): calibration and re-classification. Nephrol Dial Transplant 2010, 25(5). 10.1093/ndt/gfq046 20167948

[31] Manuel D, Maaten S, Rosella L, Wilson S, Ho T. Modelling potential impact of interventions for diabetes prevention, early detection and management: final report In: ICES Investigative Report. Toronto: Institute for Clinical Evaluative Sciences; 2008.

[32] Concordance for survival time data: fixed and time-dependent covariates and possible ties in predictor and time [http://cancercenter.mayo.edu/mayo/research/biostat/upload/80.pdf]

[33] Bouwmeester W, Zuithoff NP, Mallett S, Geerlings MI, Vergouwe Y, Steyerberg EW et al Reporting and methods in clinical prediction research: a systematic review. PLoS Med 2012, 9(5):1–12. 10.1371/journal.pmed.1001221 PMC3358324 22629234

[34] World Health Organization: Prevention of cardiovascular disease: guidelines for assessment and management of cardiovascular risk: World Health Organization; 2007.

[35] Lloyd-Jones DM, Hong Y, Labarthe D, Mozaffarian D, Appel LJ, Van Horn L et al: Defining and Setting National Goals for Cardiovascular Health Promotion and Disease Reduction: The American Heart Association’s Strategic Impact Goal Through 2020 and Beyond. Circulation 2010, 121(4):586–613. 10.1161/CIRCULATIONAHA.109.192703 20089546

[36] Steyerberg EW. Clinical Prediction Models: A Practical Approach to Development, Validation, and Updating. London: Springer; 2009.

[37] Qiao Q, Gao W, Laatikainen T, Vartiainen E. Layperson-oriented vs. clinical-based models for prediction of incidence of ischemic stroke: National FINRISK Study. International journal of stroke: official journal of the International Stroke Society 2012, 7(8):662–668. 22098944 10.1111/j.1747-4949.2011.00692.x

[38] Gaziano TA, Young CR, Fitzmaurice G, Atwood S, Gaziano JM. Laboratory-based versus non-laboratory-based method for assessment of cardiovascular disease risk: the NHANES I Follow-up Study cohort. The Lancet 2008, 371(9616):923–931. 10.1016/S0140-6736(08)60418-3 PMC2864150 18342687

[39] Noble D, Mathur R, Dent T, Meads C, Greenhalgh T. Risk models and scores for type 2 diabetes: systematic review. BMJ 2011, 343:d7163 10.1136/bmj.d7163 22123912 PMC3225074

[40] Muggah E, Graves E, Bennett C, Manuel DG. Ascertainment of chronic diseases using population health data: a comparison of health administrative data and patient self-report. BMC Public Health 2013, 13(16). 10.1186/1471-2458-13-16 PMC3557162 23302258

[41] Wong SL, Shields M, Leatherdale S, Malaison E, Hammond D. Assessment of validity of self-reported smoking status. Health reports / Statistics Canada, Canadian Centre for Health Information = Rapports sur la sante / Statistique Canada, Centre canadien d'information sur la sante 2012, 23(1):47–53. 22590805

[42] Shields M, Connor Gorber S, Tremblay MS. Estimates of obesity based on self-report versus direct measures. Health reports / Statistics Canada, Canadian Centre for Health Information = Rapports sur la sante / Statistique Canada, Centre canadien d'information sur la sante 2008, 19(2):61–76. 18642520

[43] Reynolds K, Lewis B, Nolen JD, Kinney GL, Sathya B, He J. Alcohol consumption and risk of stroke: a meta-analysis. Jama 2003, 289(5):579–588. 12578491 10.1001/jama.289.5.579

[44] Garriguet D, Colley RC. A comparison of self-reported leisure-time physical activity and measured moderate-to-vigorous physical activity in adolescents and adults. Health reports / Statistics Canada, Canadian Centre for Health Information = Rapports sur la sante / Statistique Canada, Centre canadien d'information sur la sante 2014, 25(7):3–11. 25029491

[45] Mozaffarian D, Appel LJ, Van Horn L. Components of a cardioprotective diet: new insights. Circulation 2011, 123(24):2870–2891. 10.1161/CIRCULATIONAHA.110.968735 21690503 PMC6261290

[46] Garriguet D. Diet quality in Canada. Health Reports 2009, 20(3):41–52. 19813438

[47] Manuel DG, Perez R, Bennett C, Rosella L, Taljaard M, Roberts M et al: Seven More Years: The impact of smoking, alcohol, diet, physical activity and stress on health and life expectancy in Ontario In: An ICES/PHO Report. Toronto: Institute for Clinical Evaluative Sciences and Public Health Ontario; 2012.

[48] Manuel DG, Perez R, Bennett C, Rosella L, Choi B. 900,000 Days in Hospital: The Annual Impact of Smoking, Alcohol, Diet, and Physical Activity on Hospital Use in Ontario. In. Toronto, ON: Institute for Clinical Evaluative Sciences; 2014.

[49] Collins GS, Reitsma JB, Altman DG, Moons KG. Transparent reporting of a multivariable prediction model for individual prognosis or diagnosis (TRIPOD): the TRIPOD statement. BMJ 2015, 350:g7594 10.1136/bmj.g7594 25569120

[50] Taljaard M, Tuna M, Bennett C, Perez R, Rosella L, Tu JV et al: Cardiovascular Disease Population Risk Tool (CVDPoRT): predictive algorithm for assessing CVD risk in the community setting. A study protocol. BMJ open 2014, 4(10):e006701 10.1136/bmjopen-2014-006701 25341454 PMC4208046

