# Supplementary figures and images for: Predicting Stroke Risk Based on Health Behaviours: Development of the Stroke Population Risk Tool (SPoRT)
Source: PLoS One. 2015 Dec 4;10(12):e0143342. doi: 10.1371/journal.pone.0143342 (PMC4670216; doi:10.1371/journal.pone.0143342)

**S1 Fig. Males: 5-year risk of hospitalized stroke based on behavioral and other risk factors.**


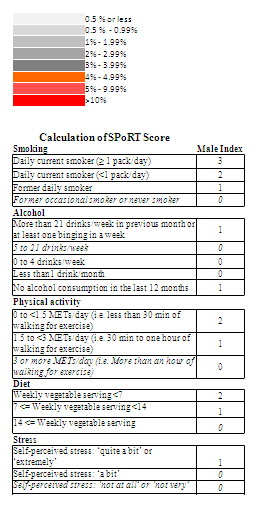

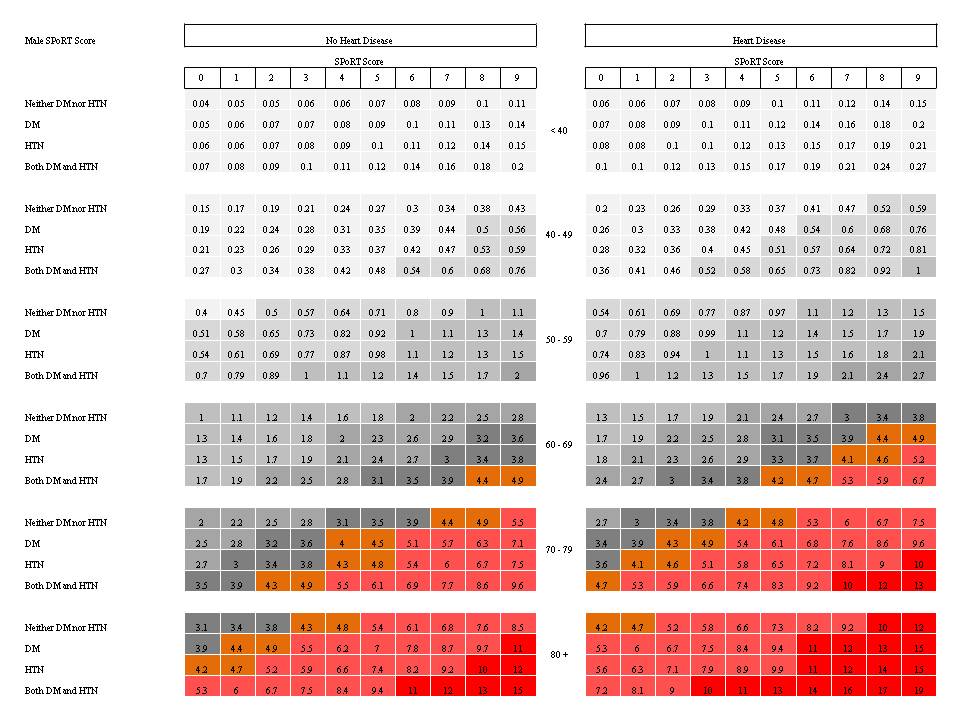

Supplement: S1 Fig — (DOCX) [file pone.0143342.s001.docx]

**S2 Fig. Females: 5-year risk of hospitalized stroke based on behavioral and other risk factors.**


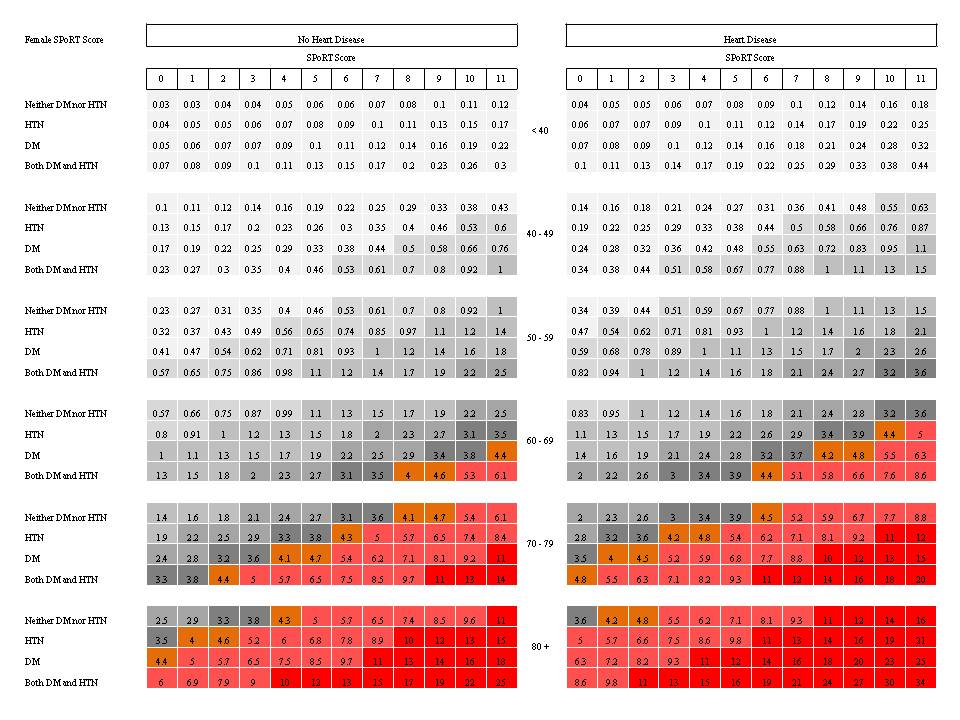

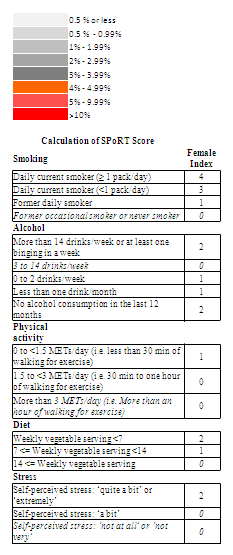

Supplement: S2 Fig — (DOCX) [file pone.0143342.s002.docx]
