# Supplementary material for: Predicting Stroke Risk Based on Health Behaviours: Development of the Stroke Population Risk Tool (SPoRT)
Source: PLoS One. 2015 Dec 4;10(12):e0143342. doi: 10.1371/journal.pone.0143342 (PMC4670216; doi:10.1371/journal.pone.0143342)
Supplement: S1 Table — (DOCX) [file pone.0143342.s003.docx]

**S1Table. Definitions for exposure variables**

| **Concept** | **CCHS survey questions for exposure variables** | **Type** |
| --- | --- | --- |
| Smoking | How many cigarettes do you smoke each day now? | Collected |
|  | On the days that you do smoke, about how many cigarettes do you usually have? | Collected |
|  | Type of smoker | Derived |
|  | In your lifetime, have you smoked a total of 100 or more cigarettes? (about 4 packs) | Collected |
|  | Have you ever smoked a whole cigarette? | Collected |
|  | At the present time do you smoke cigarettes daily, occasionally or not at all? | Collected |
|  | Have you ever smoked cigarettes daily? | Collected |
| Alcohol | Have you ever had a drink? | Collected |
|  | During the past 12 months, how often did you drink alcoholic beverages? | Collected |
|  | How often in the past 12 months have you had 5 or more drinks on one occasion? | Collected |
|  | Number of drinks - past week | Derived |
|  | During the past 12 months, have you had a drink of beer, wine, liquor or any other alcoholic beverage? | Collected |
|  | Starting with yesterday, that is [day name], how many drinks did you have? - Sunday | Collected |
|  | Starting with yesterday, that is [day name], how many drinks did you have? - Monday | Collected |
|  | Starting with yesterday, that is [day name], how many drinks did you have? -Tuesday | Collected |
|  | Starting with yesterday, that is [day name], how many drinks did you have? - Wednesday | Collected |
|  | Starting with yesterday, that is [day name], how many drinks did you have? - Thursday | Collected |
|  | Starting with yesterday, that is [day name], how many drinks did you have? - Friday | Collected |
|  | Starting with yesterday, that is [day name], how many drinks did you have? - Saturday | Collected |
| Diet | Daily consumption - carrots | Derived |
|  | Eats carrots - number of times per unit of time | Collected |
|  | Daily consumption - other vegetables | Derived |
|  | Eats other vegetables -number of times per unit of time | Collected |
| Physical Activity | Physical activity index | Derived |
|  | Activities / last 3 months | Collected |
| Stress | Thinking about the amount of stress in your life, would you say that most days are: (not at all stressful, not very stressful, a bit stressful, quite a bit stressful, or extremely stressful)? | Collected |
| Heart Disease | Do you have heart disease? | Collected |
| Diabetes | Do you have diabetes? | Collected |
| High Blood Pressure | Do you have high blood pressure? | Collected |
| Stroke | Do you suffer from the effects of a stroke? | Collected |
| Education | Highest level of education - Household | Derived |
|  | Highest level of education - respondent | Derived |
|  | What is the highest grade of elementary or high school you have ever completed? | Collected |
|  | Did you graduate from high school (secondary school)? | Collected |
|  | Did you graduate from high school (secondary school)? | Collected |
|  | Have you received any other education that could be counted towards a degree, certificate or diploma from an educational institution? | Collected |
|  | What is the highest degree, certificate or diploma you have obtained? | Collected |
| Income | Total household income from all sources | Derived |
|  | Was the total household income from all sources less than $5,000 or $5,000 or more? | Collected |
|  | Was the total household income from all sources less than $10,000 or $10,000 or more? | Collected |
|  | Was the total household income from all sources less than $15,000 or $15,000 or more? | Collected |
|  | Was the total household income less than $20,000 or $20,000 or more? | Collected |
|  | Was the total household income from all sources less than $40,000 or $40,000 or more? | Collected |
|  | Was the total household income from all sources less than $30,000 or $30,000 or more? | Collected |
|  | Was the total household income from all sources: | Collected |
|  | 1 … less than $50,000? |  |
|  | 2 … $50,000 to less than $60,000? |  |
|  | 3 … $60,000 to less than $80,000? |  |
|  | 4 … $80,000 to less than $100,000? |  |
|  | 5 … $100,000 or more? |  |
| BMI | Body Mass Index (BMI) / self-report | Derived |
|  | Height (metres) / self-reported | Derived |
|  | How tall are you without shoes on? | Collected |
|  | Weight (kilograms) / self-reported | Derived |
|  | How much do you weigh? | Collected |
| Ethnicity | Cultural or racial origin | Derived |
|  | People living in Canada come from many different cultural and racial backgrounds. Are you: | Collected |
|  | 1 …White? |  |
|  | 2 …Chinese? |  |
|  | 3 …South Asian (e.g., East Indian, Pakistani, Sri Lankan)? |  |
|  | 4 …Black? |  |
|  | 5 …Filipino? |  |
|  | 6 …Latin American? |  |
|  | 7 …Southeast Asian (e.g., Cambodian, Indonesian, Laotian, Vietnamese)? |  |
|  | 8 …Arab? |  |
|  | 9 …West Asian (e.g., Afghan, Iranian)? |  |
|  | 10 …Japanese? |  |
|  | 11 …Korean? |  |
|  | 12 …Aboriginal (North American Indian, Métis or Inuit)? |  |
|  | 13 Other - Specify |  |
