# Supplementary material for: Predicting Stroke Risk Based on Health Behaviours: Development of the Stroke Population Risk Tool (SPoRT)
Source: PLoS One. 2015 Dec 4;10(12):e0143342. doi: 10.1371/journal.pone.0143342 (PMC4670216; doi:10.1371/journal.pone.0143342)
Supplement: S2 Table — (DOCX) [file pone.0143342.s004.docx]

**S2 Table. Checklist for reporting clinical prediction research***

| **Topic** | **Item** | **SPoRT** |
| --- | --- | --- |
| **Study Design** | Type of prediction study | Prediction model development with external validation |
|  | Participant sampling or selection method | Prospective cohort |
| **Participants** | Participant recruitment | Based on a population based survey which used multistage stratified cluster design |
|  | Participation rate | CCHS 1.1 = 84.7%  CCHS 2.1 = 80.7%  CCHS 3.1 = 78.9% CCHS 4.1 = 77.6%  Average = 80.5% |
|  | Inclusion/exclusion criteria | The CCHS included all community-dwelling Canadian population 12 years of age and over living in the ten provinces and the three territories and excluded: persons living on reserves and other Aboriginal settlements in the provinces; full-time members of the Canadian Forces; institutionalized population; and persons living in remote regions. Altogether, these exclusions represent less than 3% of the target population. Further study exclusions are stated in methods section under *SPoRT derivation cohort*. |
|  | Description of cohort characteristics | See Table 1 |
|  | Study recruitment dates | 2001 to 2005 for development and 2007-2008 for validation cohort |
|  | Length of follow-up | 6.5 - 10.5 years for development / 3 - 5 years for validation |
|  | Setting | General population |
| **Candidate predictors** | Clear definition to ensure reproducibility | See Table 2 and eTable 1 |
|  | Assessment blinded for outcome | Not applicable - prospective study design |
|  | Predictor part of outcome | No |
|  | Interaction of predictors tested | We calculated power considering all interaction terms (see below). Due to a large number of potential interaction terms we focused our attention on interactions previously described in epidemiology literature, or had the potential to have an important influence on predictive accuracy of behavioural risks. For this reason, we focused our attention on age and behavioural risks as potential interactions. |
|  | Handling of continuous predictors described | Age maintained as continuous variable. Most other variables maintained their categories as ascertained in the health survey (i.e., not dichotomized). |
| **Outcome** | Clear definition to ensure reproducibility | Incident stroke. Primary outcome = hospitalized stroke; secondary outcome (i) = hospitalized ischemic stroke; (ii) stroke or TIA resulting in hospitalization or death; (iii) stroke or TIA diagnosed in the community setting by a physician or resulting in hospitalization. |
|  | Type of outcome described | ICD9 and 10 codes provided. Based on Canadian standard definition. Outcomes were ascertained using routinely-collected data (rather than active follow-up of survey respondents). There was a high agreement of stroke ascertainment of hospitalized stroke (92%) with differences attributed to misclassification of stroke type but not stroke event. We, therefore, expect only minimal bias in overall stroke estimation. We expect misclassification error of community diagnosed stroke will result in an underestimate of stroke and TIA of approximately 3.3%. This estimate is based on the difference of stroke prevalence using the ascertainment approach versus a reference standard chart review (3.1 % versus 3.0%). See Tu et al. 2013. Note that the expected bias is much smaller than expected by examined by either sensitivity or specificity alone (68.0 % and 98.9% respectfully) because the underascertained expected from imperfect sensitivity is largely counteracted by the overascertainement expected from imperfect specificity. See Manuel et al. 2010 for calculation methods. |
|  | Method of analysis of outcome | Variables maintained their original form. Several less common approaches used to improve model performance including, time-vary age, competing risk analyses, development of behavioural index. |
|  | Assessment blinded for predictors | Outcomes we coded in separately collected databases and then linked to the Canadian Population Health Survey. Predictors were preselected (not blinded) based on epidemiology evidence of causal association with stroke. |
|  | Selective reporting of outcomes (in the case of prediction of more than one outcome) | Reporting of algorithm and performance for all outcomes (i.e., primary and secondary outcome). Additional secondary outcomes were added in response to comments of reviewers of an earlier draft. |
| **Statistical power** | Effective sample size | 1551 (709 males and 842 females) events in primary analyses of derivation population. For time-to-event modelling, the effective sample size is the number of events. N/p ratios of 10 are frequently recommended for the development of stable models. (n=number of cases, p=number of variables). The number of variables in the model is 10 (age (2-3), risk behaviour index, high blood pressure (2), diabetes, heart disease, years (2). The effective sample size considering only the predictors in the final model is 70.9 for males and 84.2 for females. The effective sample size considering all candidate predictors is 16.48 for males and 19.58 for females. |
| **Selection of predictors** | Selection of predictors for inclusion in the multivariable analysis not based on statistical analysis | Predictor variables selected based on epidemiology literature. Behavioural index was used to increase statistical power of behaviour risk predictor variables thereby allowing inclusion of all behavioural risk with multiple levels of exposure. |
|  | Method of predictor selection used within multivariable analysis | Forward selection was used based on categories of predictor variable as defined by Cecchini: age, behavioural risks, sociodemographic risks, intermediate risks (BMI), proximal risks (hypertension, lipid therapy), diseases (diabetes, heart disease). |
|  | Criterion for predictor inclusion | Predictors were included if there was statistical significance p<0.05 and improvement in calibration <20% difference in observed versus predicted for any preselected subgroup with greater than 5% of total outcomes. |
| **Handling of missing values** | Reporting of missing values per predictor, or number or percentage of participants with missing values | For the most part, missing variables were less than 2% (see Table 1). The highest missing data was for income (4.9%). |
|  | Reporting of procedures for dealing with missing values | Yes. Imputed using the subgroup mean of the index value for that person’s age, sex and local health region |
| **Presentation of Results** | Reporting of univariable and multivariable predictor-outcome effects | See Methods *Model development*. Univariable reporting for main predictors are reported in eTable 7 |
|  | Reporting of full or final model | Table 3 and Table 4 |
| **Model performance measures and validation** | Type of predictive performance measures reported | Discrimination measures: C-statistics, 75:25, 95:5 ratios. Calibration measures focused on observed and predicted differences for 60+ predefined subgroups. Model performance was considered acceptable if difference was less than 20 percent for any subgroup that represented greater than 5 percent of outcomes.  The Hosmer-Lemeshow test of heterogeneity was not used due to concerns about using a test of differences when there are a large number of observed events.^1^  Reclassification differences were not a focus of the study and so were not assessed. |
|  | Type of validation | Both internal and external validation performed. |

*Adapted from: Bousmeester et al.[^24^](#_ENREF_24)

^1^ Kramer AA, Zimmerman JE. Assessing the calibration of mortality benchmarks in critical care: The Hosmer-Lemeshow test revisited *Critical Care Medicine.* 2007;35(9).
