# Supplementary material for: Predicting Stroke Risk Based on Health Behaviours: Development of the Stroke Population Risk Tool (SPoRT)
Source: PLoS One. 2015 Dec 4;10(12):e0143342. doi: 10.1371/journal.pone.0143342 (PMC4670216; doi:10.1371/journal.pone.0143342)
Supplement: S3 Table — (DOCX) [file pone.0143342.s005.docx]

**S3 Table. Baseline characteristics of the derivation (CCHS 1.1 – CCHS 3.1) and validation (CCHS 4.1) cohorts**

|  | | **Derivation Cohort** | | **Validation Cohort** | |
| --- | --- | --- | --- | --- | --- |
| **Characteristic^a^** | | **Male (n=37483)** | **Female (n=44776)** | **Male (n=13032)** | **Female (n=15573)** |
| Age, mead (SD) | | 48.2 (16.2) | 49.4 (17.1) | 50.2 (16.5) | 51.0 (17.0) |
| Smoking status | |  |  |  |  |
|  | Heavy smoker | 4499 (12.0) | 3051 (6.8) | 1289 (9.9) | 793 (5.1) |
|  | Light smoker | 5990 (16.0) | 7510 (16.8) | 2000 (15.4) | 2432 (15.6) |
|  | Former smoker | 12154 (32.4) | 10797 (24.1) | 4212 (32.3) | 3744 (24.7) |
|  | Non-smoker | 14785 (39.4) | 23359 (52.2) | 5501 (42.2) | 8479 (54.5) |
|  | Missing | 55 (0.2) | 59 (0.1) | 30 (0.2) | 26 (0.1) |
| Alcohol | |  |  |  |  |
|  | Heavy drinker | 4895 (13.1) | 1586 (3.5) | 1784 (13.7) | 665 (4.3) |
|  | Moderate drinker | 9640 (25.7) | 9900 (22.1) | 3403 (26.2) | 6681 (23.4) |
|  | Light drinker | 12877 (34.4) | 14282 (31.9) | 4360 (33.5) | 4878 (31.3) |
|  | Occasional drinker | 4106 (11.0) | 8698 (19.4) | 1325 (10.2) | 2862 (18.4) |
|  | Current non-drinker | 5403 (14.4) | 9888 (22.1) | 1856 (14.2) | 3322 (21.3) |
|  | Missing | 562 (1.5) | 422 (0.9) | 294 (2.3) | 204 (1.3) |
| Physical Activity | |  |  |  |  |
|  | Inactive | 17216 (45.9) | 23270 (52.0) | 6028 (46.3) | 8113 (52.1) |
|  | Moderately active | 9177 (24.5) | 11485 (25.7) | 3284 (25.2) | 3986 (25.6) |
|  | Active | 9846 (26.3) | 9448 (21.1) | 3509 (26.9) | 3357 (21.6) |
|  | Missing | 1244 (3.3) | 537 (1.3) | 211 (1.6) | 117 (0.8) |
| Diet | |  |  |  |  |
|  | Poor diet | 11769 (31.4) | 8627 (19.3) | 3953 (30.3) | 2886 (18.5) |
|  | Fair diet | 15810 (42.2) | 17606 (39.3) | 5009 (38.4) | 5327 (34.2) |
|  | Adequate diet | 8968 (23.9) | 17825 (39.8) | 3563 (27.3) | 6937 (44.6) |
|  | Missing | 936 (2.5) | 718 (1.6) | 507 (3.9) | 423 (2.7) |
| Self perceived stress | |  |  |  |  |
|  | High stress | 8162 (21.8) | 10721 (23.9) | 2498 (19.2) | 3314 (21.3) |
|  | Low stress | 29256 (78.1) | 33988 (75.9) | 10487 (80.5) | 12186 (78.3) |
|  | Missing | 65 (0.2) | 67 (0.2) | 47 (3.9) | 73 (0.5) |
| Highest family education | |  |  |  |  |
|  | < Secondary school | 4093 (10.9) | 6286 (14.0) | 1164 (8.9) | 1659 (10.7) |
|  | Secondary school | 5170 (13.8) | 6321 (20.4) | 1553 (11.9) | 1896 (12.2) |
|  | Some post-secondary | 2218 (5.9) | 2659 (7.1) | 645 (5.0) | 756 (4.9) |
|  | Post-secondary | 24702 (65.9) | 28396 (51.4) | 9044 (69.4) | 10743 (69.0) |
|  | Missing | 1300 (3.5) | 1114 (0.7) | 626 (4.8) | 519 (3.3) |
| History of hypertension | |  |  |  |  |
|  | Yes | 6950 (18.5) | 9232 (20.6) | 2933 (22.5) | 3721 (23.9) |
|  | No | 30455 (81.3) | 35503 (79.3) | 10042 (77.1) | 11827 (75.9) |
|  | Missing | 78 (0.2) | 41 (0.1) | 57 (0.4) | 25 (0.2) |
| History of heart disease | |  |  |  |  |
|  | Yes | 3009 (8.0) | 2884 (6.4) | 1047 (8.0) | 960 (6.2) |
|  | No | 34434 (91.9) | 41842 (93.5) | 11949 (91.7) | 14581 (93.6) |
|  | Missing | 40 (0.1) | 51 (0.1) | 36 (0.3) | 32 (0.2) |
| History of diabetes | |  |  |  |  |
|  | Yes | 2625 (7.0) | 2659 (5.9) | 1196 (9.2) | 1135 (7.3) |
|  | No | 34838 (92.9) | 42094 (94.0) | 11819 (90.7) | 14428 (92.6) |
|  | Missing | 20 (0.1) | 23 (0.1) | 17 (0.1) | 10 (0.1) |
|  |  |  |  |  |  |
| Abbreviations: CCHS = Canadian Community Health Survey; HBP = high blood pressure; SD = standard deviation.  ^a^Unless otherwise stated, values represent number and (percentage). Category descriptions are presented in Table 2.  Percentages may not sum to 100 due to rounding. | | | | | |
