# Supplementary material for: Predicting Stroke Risk Based on Health Behaviours: Development of the Stroke Population Risk Tool (SPoRT)
Source: PLoS One. 2015 Dec 4;10(12):e0143342. doi: 10.1371/journal.pone.0143342 (PMC4670216; doi:10.1371/journal.pone.0143342)
Supplement: S4 Table — (DOCX) [file pone.0143342.s006.docx]

**S4 Table. Hazard ratios for individual risk factors**

|  | | **Hazard Ratio (95% CI)** | |
| --- | --- | --- | --- |
|  | | **Male** | **Female** |
| Smoking | |  |  |
|  | Heavy smoker | 1.55 (1.18-2.04) | 2.18 (1.69-2.81) |
|  | Light smoker | 1.41(1.08-1.85) | 1.67 (1.36-2.05) |
|  | Former smoker | 1.17 (0.98-1.39) | 1.03 (0.87-1.2) |
|  | Non-smoker smoker | 1.0 [Reference] | 1.0 [Reference] |
| Alcohol | |  |  |
|  | Heavy drinker | 1.23(0.92-1.66) | 1.25 (0.73-2.17) |
|  | Moderate drinker | 1.0 [Reference] | 1.0 [Reference] |
|  | Light drinker | 0.96 (0.78-1.18) | 1.07 (0.86-1.34) |
|  | Occasional drinker | 1.11 (0.86-1.43) | 1.17 (0.93- 1.48) |
|  | Current non-drinker | 1.17(0.94-1.45) | 1.39 (1.13-1.71) |
| Physical activity | |  |  |
|  | Inactive | 1.29 (1.06-1.58) | 1.27 (1.03-1.56) |
|  | Moderately active | 1.22 (0.98-1.53) | 1.05 (0.82-1.33) |
|  | Active | 1.0 [Reference] | 1.0 [Reference] |
| Diet | |  |  |
|  | Poor diet | 1.50 (1.22-1.84) | 1.42 (1.17-1.71) |
|  | Fair diet | 1.23 (1.02-1.49) | 1.23 (1.06-1.44) |
|  | Adequate diet | 1.0 [Reference] | 1.0 [Reference] |
| Stress | |  |  |
|  | Very high stress | 1.21 (0.97-1.5) | 1.39 (1.16-1.68) |
|  | Somewhat stressed | 1.05 (0.89-1.25) | 1.05 (0.90-1.23) |
|  | Low stress | 1.0 [Reference] | 1.0 [Reference] |
