# Supplementary material for: Predicting Stroke Risk Based on Health Behaviours: Development of the Stroke Population Risk Tool (SPoRT)
Source: PLoS One. 2015 Dec 4;10(12):e0143342. doi: 10.1371/journal.pone.0143342 (PMC4670216; doi:10.1371/journal.pone.0143342)
Supplement: S5 Table — (DOCX) [file pone.0143342.s007.docx]

**S5 Table. Sensitivity Analysis**

|  |  |  | **Hospitalized Stroke** | | |  |  |
| --- | --- | --- | --- | --- | --- | --- | --- |
|  | **Death from stroke** | **Death or hospitalization from stroke** | **Ischemic stroke only** | **TIA excluded** | **All stroke hospitalizations*** | **Hospital & community stroke** |  |
| **Male Models** |  |  |  |  |  |  |  |
| Age | 1.09 (1.05 -1.13) | 1.10 (1.08 -1.12) | 1.12 (1.1 -1.14) | 1.11 (1.09 -1.13) | 1.11 (1.09 -1.12) | 1.12 (1.1 -1.13) |  |
| Age spline (65 years) | 0.98 (0.92 -1.04) | 0.97 (0.95 -1) | 0.96 (0.93 -0.98) | 0.97 (0.95 -0.99) | 0.97 (0.95 -0.99) | 0.96 (0.95 -0.98) |  |
| Age time varying (per year) | 1.006 (0.991 -1.009) | 0.999 (0.997 -1.003) | 0.997 (0.9939- 0.999) | 0.997 (0.995- 0.9997) | 0.998 (0.9955- 0.9998) | 0.996 (0.994 -0.997) |  |
| SPoRT Score (per unit [0-9]) | 1.22 (1.07 -1.38) | 1.17 (1.16 -1.18) | 1.16 (1.1 -1.21) | 1.14 (1.09 -1.2) | 1.14 (1.1 -1.19) | 1.09 (1.06 -1.12) |  |
| Hypertension |  |  |  |  |  |  |  |
| No | 1.0 [Reference] | 1.0 [Reference] | 1.0 [Reference] | 1.0 [Reference] |  | 1.0 [Reference] |  |
| Yes | 1.16 (0.72 -1.88) | 1.33 (1.3 -1.35) | 1.42 (1.19 -1.7) | 1.4 (1.19 -1.65) | 1.36 (1.17 -1.58) | 1.24 (1.12 -1.37) |  |
| Missing | 0 (0 -0) | 1.61 (0.389 -6.653) | 1.65 (0.4 -6.83) | 1.41 (0.34 -5.79) | 1.14 (0.28 -4.69) | 1.6 (0.7 -3.66) |  |
| Heart Disease |  |  |  |  |  |  |  |
| No | 1.0 [Reference] | 1.0 [Reference] | 1.0 [Reference] | 1.0 [Reference] | 1.0 [Reference] | 1.0 [Reference] |  |
| Yes | 1.02 (0.58 -1.77) | 1.35 (1.12 -1.63) | 1.34 (1.09 -1.63) | 1.28 (1.06 -1.54) | 1.37 (1.16 -1.62) | 1.24 (1.1 -1.4) |  |
| Diabetes |  |  |  |  |  |  |  |
| No | 1.0 [Reference] | 1.0 [Reference] | 1.0 [Reference] | 1.0 [Reference] | 1.0 [Reference] | 1.0 [Reference] |  |
| Yes | 1.35 (0.77 -2.38) | 1.55 (1.27 -1.89) | 1.57 (1.27 -1.94) | 1.38 (1.13 -1.69) | 1.42 (1.18 -1.69) | 1.25 (1.1 -1.42) |  |
| Survey cycle |  |  |  |  |  |  |  |
| 4.1 (2007) | 1.76 (0.67 -4.62) | 0.8 (0.57 -1.12) | 0.73 (0.55 -0.97) | 0.71 (0.54 -0.93) | 0.73 (0.57 -0.93) | 0.67 (0.57 -0.79) |  |
| 3.1 (2005) | 1.0 [Reference] | 1.0 [Reference] | 1.0 [Reference] | 1.0 [Reference] | 1.0 [Reference] | 1.0 [Reference] |  |
| 2.1 (2003) | 1.99 (0.91 -4.35) | 1.05 (0.83 -1.32) | 0.96 (0.76 -1.21) | 1.05 (0.85 -1.3) | 1.04 (0.85 -1.27) | 1.02 (0.89 -1.17) |  |
| 1.1 (2001) | 2.47 (1.15 -5.33) | 1.38 (1.1 -1.73) | 1.19 (0.95 -1.5) | 1.32 (1.07 -1.63) | 1.34 (1.1 -1.62) | 1.2 (1.06 -1.37) |  |
| **Model Assessment** |  |  |  |  |  |  |  |
| Discrimination |  |  |  |  |  |  |  |
| C-stat (95% CI) | 0.84 ( 0.80-0.88) | 0.85 (0.83 - 0.86) | 0.86 ( 0.84 - 0.87) | 0.85 (0.83 -0.86) | 0.85 (0.84 - 0.86) | 0.84 (0.83 - 0.85) |  |
| Ratio of 75 to 25 risk percentile | 13.4 | 12.9 | 16.8 | 13.1 | 13.4 | 13.3 |  |
| Ratio of 95 to 5 risk percentile | 194.8 | 207.7 | 215.9 | 150.9 | 155.9 | 142.4 |  |
| Calibration |  |  |  |  |  |  |  |
| Subgroup differences^b^ No. (%) | - | 2 (2.9) | 3 (4.5) | 4 (5.9) | 1 (1.5) | 1 (1.4) |  |
| **Female Models** | |  |  |  |  |  |  |
| Age | | 1.16 (1.11-1.21) | 1.11 (1.091 - 2.12) | 1.12 (1.1 , 1.13) | 1.11 (1.09 , 1.12) | 1.1 (1.09 , 1.12) | 1.09 (1.08 , 1.1) |
| Age time varying (per year) | | 0.992 (0.986 - 0.999) | 0.996(0.993 - 0.999) | 0.996 (0.9936 , 0.9986) | 0.996 (0.9943 , 0.9985) | 0.996 (0.9942 , 1.9761) | 0.997 (0.996 , 0.998) |
| SPoRT Score (per unit [0-11]) | | 1.06 (0.96 - 1.17) | 1.16 (1.11 - 1.20) | 1.18 (1.13 , 1.22) | 1.17 (1.13 , 1.21) | 1.15 (1.13 , 1.21) | 1.11 (1.09 , 1.14) |
| Hypertension | |  |  |  |  |  |  |
| No | | 1.0 [Reference] | 1.0 [Reference] | 1.0 [Reference] |  | 1.0 [Reference] | 1.0 [Reference] |
| Yes | | 1.5 (0.999 - 2.26) | 1.49 (1.27 - 1.75) | 1.44 (1.22 , 1.7) | 1.39 (1.2 , 1.62) | 1.39 (1.2 , 1.62) | 1.28 (1.16 , 1.41) |
| Missing | | 12.09 (1.99 - 73.5) | 1.77 (0.28 - 10.90) | 1.85 (0.28 , 12.04) | 1.47 (0.23 , 9.57) | 1.18 (0.23 , 9.57) | - |
| Heart Disease | |  |  |  |  |  |  |
| No | | 1.0 [Reference] | 1.0 [Reference] | 1.0 [Reference] |  | 1.0 [Reference] | 1.0 [Reference] |
| Yes | | 1.12 (0.69 - 1.81) | 1.42 (1.18 -1.70) | 1.36 (1.13 , 1.64) | 1.32 (1.1 , 1.57) | 1.37 (1.1 , 1.57) | 1.36 (1.21 , 1.52) |
| Diabetes | |  |  |  |  |  |  |
| No | | 1.0 [Reference] | 1.0 [Reference] | 1.0 [Reference] |  | 1.0 [Reference] | 1.0 [Reference] |
| Yes | | 1.46 (0.89 - 2.45) | 1.83 (1.52 - 2.22) | 1.89 (1.56 , 2.3) | 1.73 (1.44 , 2.08) | 1.74 (1.44 , 2.08) | 1.45 (1.28 , 1.64) |
| Survey cycle | |  |  |  |  |  |  |
| 4.1 (2007) | | 1.11 (0.5 - 2.46) | 0.69 (0.50 - 0.96) | 0.744 (0.555 , 0.997) | 0.74 (0.57 , 0.96) | 0.75 (0.57 , 0.96) | 0.74 (0.63 , 0.86) |
| 3.1 (2005) | | 1.0 [Reference] | 1.0 [Reference] | 1.0 [Reference] | 1.0 [Reference] | 1.0 [Reference] | 1.0 [Reference] |
| 2.1 (2003) | | 1.19 (0.67 - 2.12) | 1.05 (0.843 - 1.30) | 1.20 (0.96 , 1.49) | 1.10 (0.9 , 1.35) | 1.05 (0.9 , 1.35) | 1.11 (0.98 , 1.26) |
| 1.1 (2001) | | 1.4 (0.81 -2.42) | 1.20 (0.97 - 1.48) | 1.36 (1.1 , 1.69) | 1.24 (1.02 , 1.51) | 1.22 (1.02 , 1.51) | 1.25 (1.1 , 1.41) |
| **Model Assessment** | |  |  |  |  |  |  |
| Discrimination | |  |  |  |  |  |  |
| C-stat (95% CI) | | 0.90 (0.87-0.92) | 0.87 (0.86 - 0.88) | 0.88 (0.87 - 0.90) | 0.87 (0.85 - 0.88) | 0.86 (0.85 - 0.88) | 0.84 (0.83 - 0.85) |
| Ratio of 75- 25 risk percentile | | 34.2 | 15.2 | 18.3 | 14.2 | 14 | 10.4 |
| Ratio of 95 -5 risk percentile | | 946.6 | 271.9 | 317 | 192.2 | 181.4 | 101.3 |
| Calibration | |  |  |  |  |  |  |
| Subgroup differences^b^ No. (%) | | - | 1 (1.6) | 2 (3.1) | 2 (3.2) | 3 (4.6) | 5 (7.6) |

*Primary study outcome – combined derivation and validation datasets
