# Supplementary material for: Predicting Stroke Risk Based on Health Behaviours: Development of the Stroke Population Risk Tool (SPoRT)
Source: PLoS One. 2015 Dec 4;10(12):e0143342. doi: 10.1371/journal.pone.0143342 (PMC4670216; doi:10.1371/journal.pone.0143342)
Supplement: S6 Table — (DOCX) [file pone.0143342.s008.docx]

**S6 Table. Comparison of SPoRT to Ideal Cardiovascular Health, developed by the American Heart Association.**

|  | **SPoRT algorithm** | **Ideal Cardiovascular Health** |
| --- | --- | --- |
| **Risk factors^a^** |  |  |
| Health behaviours  (Proximal risks) | Smoking  Diet  Physical activity  Alcohol  Stress | Smoking  Diet^b^  Physical activity |
| Clinical and laboratory  (Intermediary and distal risks) | *Ascertained using self-reports*  Body Mass Index  Hypertension  Diabetes | *Ascertained using physical measures*  Body Mass Index  Blood pressure  Fasting plasma glucose  Total cholesterol |
| Sociodemographic risk factors | Age  Sex | Not applicable |
| **Risk factor scoring** |  |  |
| Risk factor value (for each risk factor) | 0 to 4  Varies for each risk factor depending on hazard. | Binary value (yes, no)  Based on meeting healthy definition. |
| Summary score | 5-year risk (%), or  Risk categories (e.g., > 10%). | Score from 1 (ideal) to 7 |
| **Target population** | General population of adults without cardiovascular disease. | Same, except children included |

^a^ SPoRT uses self-reports for all measures, Ideal Cardiovascular Health uses both self-reported and clinical/physical measures. E.g., “have you been diagnosed with blood pressure (SPoRT) versus blood pressure <120/<80 mmHg (Ideal Cardiovascular Health).

^b^ Ideal Cardiovascular Health measures healthy diet score using 5 aspects of diet.
