# Supplementary material for: Predicting Stroke Risk Based on Health Behaviours: Development of the Stroke Population Risk Tool (SPoRT)
Source: PLoS One. 2015 Dec 4;10(12):e0143342. doi: 10.1371/journal.pone.0143342 (PMC4670216; doi:10.1371/journal.pone.0143342)
Supplement: S7 Table — (DOCX) [file pone.0143342.s009.docx]

**S7 Table. SPoRT formula**

Sport Formula

Male:

H(t)=∑ _t_ h_0_(t)* [0.105 * Age+ (-0.032)* Age_65_spline

+ ( 0.116)* Risk Behavior Index

+ (0.312) * HTN + (-0.223)*HTN_missing + (0.254)*DM

+ (0.309)*Heart Disease + (0.234)*surveycycle1+( 0.032)*surveycycle2 +(-7.62e-06)*age *t])

Risk= 1-exp(-H(t))

Female:

H(t)= ∑ _t_ h_0_(t)* [0.101 * Age

+ (0.137)* Risk Behavior Index

+ (0.328) *HTN+ (0.424)*HTN_missing + (0.555)*DM+ (0.367)*Heart Disease

+ (0.169)*surveycycle1+(0.047)*surveycycle2 +(-1.19e-05)*age *t]

Risk= 1-exp(-H(t))

t : number of days until the even
