# Supplementary material for: Predicting Stroke Risk Based on Health Behaviours: Development of the Stroke Population Risk Tool (SPoRT)
Source: PLoS One. 2015 Dec 4;10(12):e0143342. doi: 10.1371/journal.pone.0143342 (PMC4670216; doi:10.1371/journal.pone.0143342)
Supplement: S8 Table — (DOCX) [file pone.0143342.s010.docx]

**S8 Table. Crude and age standardized stroke incidence rate per 10000 person-years**

|  | **Men** | | | | **Women** | | | |
| --- | --- | --- | --- | --- | --- | --- | --- | --- |
|  | **Total person-years** | **Number of incident cases** | **Crude incidence rate** | **Age standardised rates**  **(95% CI)** | **Total person-years** | **Number of incident cases** | **Crude incidence rate** | **Age standardised rates**  **(95% CI)** |
| Total | 311484 | 709 | 22.76 | - | 376837 | 842 | 22.34 | - |
| **Age (years):** |  |  |  |  |  |  |  |  |
| <30 | 44454 | 1 | 0.22 | - | 56261 | 7 | 1.24 | - |
| 30 - 39 | 67706 | 16 | 2.36 | - | 75965 | 17 | 2.24 | - |
| 40 - 49 | 67564 | 48 | 7.10 | - | 71151 | 37 | 5.20 | - |
| 50 - 59 | 56569 | 103 | 18.21 | - | 66708 | 90 | 13.49 | - |
| 60 - 69 | 43163 | 181 | 41.93 | - | 53582 | 192 | 35.83 | - |
| 70 + | 32026 | 360 | 112.41 | - | 53170 | 499 | 93.85 | - |
| **Smoking Status** |  |  |  |  |  |  |  |  |
| Heavy smoker | 38088 | 71 | 18.64 | 32.43 (23.18-44.14) | 26076 | 70 | 26.84 | 39.47 (29.20 to 52.19) |
| Light smoker | 50224 | 75 | 14.93 | 30.41 (23.08-39.32) | 63626 | 119 | 18.70 | 32.35 (26.32 to 39.34) |
| Former smoker | 97325 | 365 | 37.50 | 25.82 (23.21-28.64) | 89336 | 216 | 24.18 | 22.28 (19.40 to 25.48) |
| Non-smoker | 125398 | 196 | 15.63 | 21.53 (18.59-24.79) | 197336 | 433 | 21.94 | 21.31 (19.35 to 23.42) |
| Missing | 448 | 2 | 44.62 | 38.02 (4.1-143.07) | 462 | 4 | 86.55 | 74.46 (20.13 to 191.41) |
| **Alcohol** |  |  |  |  |  |  |  |  |
| Heavy drinker | 41105 | 60 | 14.60 | 27.81 (20.20-37.34) | 13293 | 14 | 10.53 | 28.6 (14.55 to 50.51) |
| Moderate drinker | 80426 | 182 | 22.63 | 24.01 (20.63-27.79) | 83977 | 128 | 15.24 | 18.89 (15.69 to 22.54) |
| Light drinker | 108705 | 197 | 18.12 | 22.52 (19.45-25.94) | 121900 | 206 | 16.9 | 21.48 (18.61 to 24.66) |
| Occasional drinker | 34074 | 89 | 26.12 | 26.37 (21.18-32.46) | 73980 | 162 | 21.9 | 23.77 (20.24 to 27.73) |
| Current non-drinker | 42845 | 160 | 37.34 | 29.18 (24.80-34.10) | 80345 | 324 | 40.33 | 30.22 (26.97 to 33.75) |
| Missing | 4328 | 21 | 48.52 | 38.49 (23.81-58.86) | 3340 | 8 | 23.95 | 21.67 (9.31 to 42.83) |
| **Physical Activity** |  |  |  |  |  |  |  |  |
| Inactive | 142300 | 362 | 25.44 | 28.74 (25.83-31.89) | 194898 | 540 | 27.71 | 27.19 (24.94 to 29.58) |
| Moderately active | 76650 | 172 | 22.44 | 24.88 (21.29-28.91) | 97687 | 172 | 17.61 | 19.89 (17.00 to 23.13) |
| Active | 81755 | 142 | 17.37 | 20.28 (17.06-23.93) | 79486 | 113 | 14.22 | 20.18 (16.53 to 24.38) |
| Missing | 10778 | 33 | 30.62 | 26.58 (18.18-37.51) | 4766 | 17 | 37.67 | 32.59 (18.85 to 52.47) |
| **Diet** |  |  |  |  |  |  |  |  |
| Poor diet | 99563 | 221 | 22.20 | 31.28 (27.16-35.85) | 74312 | 178 | 23.95 | 29.76 (25.52 to 34.50) |
| Fair diet | 132495 | 307 | 23.17 | 25.23 (22.47-28.23) | 149288 | 374 | 25.05 | 25.80 (23.25 to 28.55) |
| Adequate diet | 72776 | 152 | 20.89 | 20.05 (16.99-23.51) | 148009 | 265 | 17.90 | 19.65 (17.34 to 22.19) |
| Missing | 6649 | 29 | 43.62 | 28.86 (19-42.02) | 5228 | 25 | 47.82 | 28.97 (18.26 to 43.65) |
| **Self perceived stress** |  |  |  |  |  |  |  |  |
| High stress | 69482 | 108 | 15.54 | 29.72 (23.36-37.27) | 92490 | 160 | 17.3 | 30.67 (25.67 to 36.37) |
| Somewhat stressed | 127249 | 222 | 17.45 | 25.43 (22.07-29.15) | 156254 | 279 | 17.86 | 23.29 (20.61 to 26.23) |
| Low stress | 114306 | 375 | 32.81 | 24.16 (21.73-26.79) | 127582 | 402 | 31.51 | 22.62 (20.43 to 24.98) |
| Missing | 446 | 4 | 89.69 | 76.74 (11.16-259.02) | 511 | 1 | 19.56 | 11.11 (0.28 to 61.88) |
| **Education** |  |  |  |  |  |  |  |  |
| <Secondary school graduation | 31418 | 188 | 59.84 | 33.90 (28.79-39.64) | 50262 | 316 | 62.87 | 31.75 (31.04 to 40.96) |
| Secondary school graduation | 43619 | 100 | 22.93 | 24.30 (19.75-29.59) | 53961 | 129 | 23.91 | 23.08 (19.26 to 27.42) |
| Some post secondary | 18844 | 46 | 24.41 | 32.39 (23.61-43.36) | 23182 | 41 | 17.69 | 21.90 (15.67 to 29.77) |
| Post-secondary graduation | 207824 | 348 | 16.74 | 22.62 (20.24-25.19) | 240614 | 344 | 14.30 | 21.48 (19.19 to 23.97) |
| Missing | 9778 | 27 | 27.61 | 29.77 (19.62-43.31) | 8817 | 12 | 13.61 | 15.18 (7.83 to 26.54) |
| **Income** |  |  |  |  |  |  |  |  |
| <30K | 51652 | 242 | 46.85 | 34.05 (29.72-38.84) | 99569 | 426 | 42.78 | 30.34 (27.37 to 25.91) |
| 30K - < 50K | 63858 | 173 | 27.09 | 24.2 (20.71-28.12) | 75767 | 165 | 21.78 | 22.03 (18.77 to 25.69) |
| 50K - < 80K | 85237 | 143 | 16.78 | 23.55 (19.73-27.9) | 87646 | 95 | 10.84 | 20.56 (16.32 to 25.57) |
| 80K+ | 93072 | 98 | 10.53 | 20.61 (15.8-26.42) | 83038 | 56 | 6.74 | 25.31 (17.80 to 34.92) |
| Missing | 17664 | 53 | 30.00 | 24.78 (18.4-32.67) | 30818 | 100 | 32.45 | 22.79 (18.44 to 27.86) |
| **BMI** |  |  |  |  |  |  |  |  |
| <18.5 | 2548 | 5 | 19.62 | 21.66 (7.03-50.54) | 10770 | 25 | 23.21 | 31.91 (20.48 to 47.43) |
| 18.5 - <23 | 50202 | 95 | 18.92 | 24.34 (19.68-29.77) | 111654 | 203 | 18.18 | 24.16 (20.93 to 27.75) |
| 23 - < 25 | 64639 | 139 | 21.50 | 23.23 (19.52-27.43) | 65610 | 124 | 18.90 | 19.32 (16.07 to 23.04) |
| 25 - <30 | 134030 | 320 | 23.88 | 25.19 (22.50-28.12) | 107883 | 300 | 27.81 | 24.69 (21.97 to 27.66) |
| 30 - <35 | 45520 | 119 | 26.14 | 29.45 (24.15-35.57) | 42730 | 113 | 26.45 | 25.73 (21.16 to 31.00) |
| 35 + | 13248 | 28 | 21.14 | 26.62 (16.30-41.04) | 20558 | 54 | 26.27 | 33.41 (24.67 to 44.24) |
| Pregnant | - | - | - | - | 7133 | 0 | 0.00 | - |
| Missing | 1298 | 3 | 23.11 | 20.33 (4.18-59.50) | 10498 | 23 | 21.91 | 25.52 (16.10 to 38.44) |
| **Ethnicity** |  |  |  |  |  |  |  |  |
| White | 279730 | 671 | 23.99 | 25.85 (23.92-27.89) | 340415 | 805 | 23.65 | 24.39 (22.74 to 26.14) |
| Visible minority | 31475 | 36 | 11.44 | 18.07 (12.25-25.7) | 36083 | 36 | 9.98 | 21.68 (14.63 to 30.95) |
| Missing | 278 | 2 | 71.94 | 81.14 (7.57-320.2) | 339 | 1 | 29.50 | 27.00 (0.68 to 150.44) |
| **High Blood Pressure** |  |  |  |  |  |  |  |  |
| Yes | 53574 | 290 | 54.13 | 35.61 (31.09-40.6) | 72500 | 425 | 58.62 | 34.39 (29.92 to 39.35) |
| No | 257303 | 418 | 16.25 | 22.78 (20.61-25.13) | 303993 | 416 | 13.68 | 20.00 (18.07 to 22.07) |
| Missing | 607 | 1 | 16.47 | 13.29 (0.34-74.06) | 344 | 1 | 29.07 | 38.59 (0.98 to 215.03) |
| **Heart disease** |  |  |  |  |  |  |  |  |
| Yes | 21242 | 184 | 86.62 | 42.38 (35.18-50.62) | 21512 | 198 | 92.04 | 48.45 (38.38 to 60.35) |
| No | 289959 | 524 | 18.07 | 22.97 (21.01-25.06) | 354941 | 644 | 18.14 | 21.55 (19.90 to 23.30) |
| Missing | 282 | 1 | 35.46 | 16.41 (0.42-91.41) | 384 | 0 | 0.00 | - |
| **Diabetes** |  |  |  |  |  |  |  |  |
| Yes | 19187 | 136 | 70.88 | 44.1 (35.49-54.18) | 19979 | 164 | 82.09 | 51.93 (43.42 to 61.62) |
| No | 292142 | 573 | 19.61 | 23.83 (21.9-25.89) | 356673 | 678 | 19.01 | 21.78 (20.17 to 23.49) |
| Missing | 154 | 0 | 0.00 | - | 184 | 0 | 0.00 | - |
